# A Comprehensive Review on the Chemical Properties, Plant Sources, Pharmacological Activities, Pharmacokinetic and Toxicological Characteristics of Tetrahydropalmatine

**DOI:** 10.3389/fphar.2022.890078

**Published:** 2022-04-26

**Authors:** Qinyun Du, Xianli Meng, Shaohui Wang

**Affiliations:** ^1^ State Key Laboratory of Southwestern Chinese Medicine Resources, School of Pharmacy, Chengdu University of Traditional Chinese Medicine, Chengdu, China; ^2^ State Key Laboratory of Southwestern Chinese Medicine Resources, Innovative Institute of Chinese Medicine and Pharmacy, Chengdu University of Traditional Chinese Medicine, Chengdu, China; ^3^ State Key Laboratory of Southwestern Chinese Medicine Resources, School of Ethnic Medicine, Chengdu University of Traditional Chinese Medicine, Chengdu, China

**Keywords:** tetrahydropalmatine, pharmacological activity, pharmacokinetic characteristics, toxicity, chemical properties

## Abstract

Tetrahydropalmatine (THP), a tetrahydroproberine isoquinoline alkaloid, is widely present in some botanical drugs, such as *Stephania epigaea* H.S. Lo (Menispermaceae; Radix stephaniae epigaeae), *Corydalis yanhusuo* (Y.H.Chou & Chun C.Hsu) W.T. Wang ex Z.Y. Su and C.Y. Wu (Papaveraceae; Corydalis rhizoma), and *Phellodendron chinense* C.K.Schneid (Berberidaceae; Phellodendri chinensis cortex). THP has attracted considerable attention because of its diverse pharmacological activities. In this review, the chemical properties, plant sources, pharmacological activities, pharmacokinetic and toxicological characteristics of THP were systematically summarized for the first time. The results indicated that THP mainly existed in *Papaveraceae* and *Menispermaceae* families. Its pharmacological activities include anti-addiction, anti-inflammatory, analgesic, neuroprotective, and antitumor effects. Pharmacokinetic studies showed that THP was inadequately absorbed in the intestine and had rapid clearance and low bioavailability *in vivo*, as well as self-microemulsifying drug delivery systems, which could increase the absorption level and absorption rate of THP and improve its bioavailability. In addition, THP may have potential cardiac and neurological toxicity, but toxicity studies of THP are limited, especially its long-duration and acute toxicity tests. In summary, THP, as a natural alkaloid, has application prospects and potential development value, which is promising to be a novel drug for the treatment of pain, inflammation, and other related diseases. Further research on its potential target, molecular mechanism, toxicity, and oral utilization should need to be strengthened in the future.

## Introduction

**FIGURE 1:**
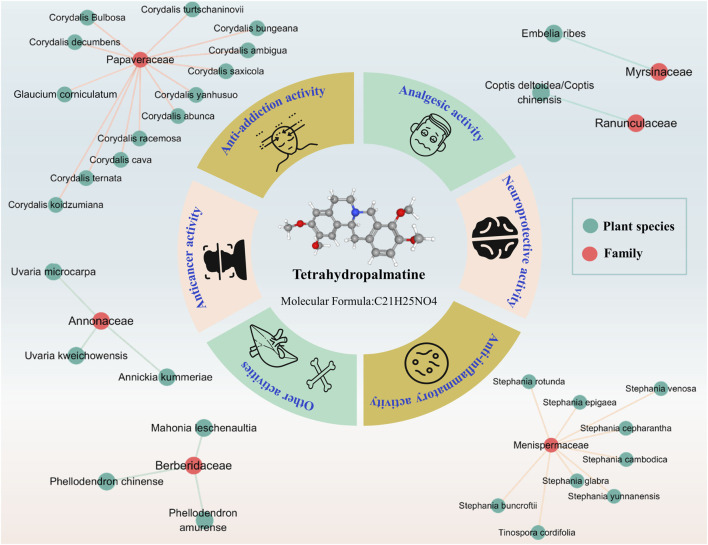
Plant source and potential pharmacological activity of THP. (References to all information specified in the figure are in the Supporting materials References).

Alkaloids, as a class of basic organic compounds containing nitrogen, widely exist in nature and have good potential biological activities and development value. Tetrahydropalmatine (THP, PubChem CID: 5417), with one chiral center, is a tetrahydroprotoberberine isoquinoline alkaloid and can be extracted from *Stephania* and *Corydalis* (Alasvand et al., 2019; Liu C. et al., 2019). Modern studies have shown that THP is an active ingredient in some common Chinese medicines, such as *Stephania epigaea* H.S. Lo [Menispermaceae; Radix stephaniae epigaeae] (Sun et al., 2020; Xiao et al., 2021), *Corydalis yanhusuo* (Y.H.Chou & Chun C.Hsu) W.T.Wang ex Z.Y.Su and C.Y.Wu [Papaveraceae; Corydalis rhizoma] (Henkes et al., 2011), and *Stephania yunnanensis* H.S. Lo [Menispermaceae; Yunnan Di Bu Rong] (Ma et al., 2008). and *Corydalis ternata* (Nakai) Nakai [Papaveraceae; Corydalis rhizoma] (Yun, 2014; Kim et al., 2017)*.* THP is also found in botanical drugs used in some Southeast Asian countries and African countries, *Stephania rotunda* Lour [Menispermaceae; Koma pich] (Baghdikian et al., 2013; Bory et al., 2013; Desgrouas et al., 2014), *Stephania venosa* (Blume) Spreng [Menispermaceae; Sa-Bu-Leud] (Kongkiatpaiboon et al., 2016; Le et al., 2017), and *Tinospora cordifolia* (Willd.) Hook.f. and Thomson [Menispermaceae; Guduchi] (Bajpai et al., 2016; Singh and Chaudhuri, 2017; Chowdhury, 2021). Hence, the pharmacological activities of THP have been extensively studied in recent years, especially its analgesic, anti-addictive, anti-inflammatory, neuroprotection, and anticancer activities (Figure 1). These studies suggested that THP is a promising compound for treating dysmenorrhea, drug addiction, inflammatory diseases, neuropathic pain, cancer, brain edema, and acute global cerebral ischemia-reperfusion injury.

In the past few decades, the pharmacological activities of THP have been widely studied, and its pharmacokinetic characteristics and toxicity have been gradually elucidated in recent years. However, numerous studies on THP are limited and lack systematic induction and summaries. Thus, in this review, the plant origin, pharmacological activity, pharmacokinetic and toxicological characteristics of THP up to 2021 were comprehensively summarized by using Pubmed (https://pubmed.ncbi.nlm.nih.gov/), Web of Science (https://www.webofscience.com/wos/woscc/basic-search), ScienceDirect (https://www.sciencedirect.com/), and CNKI (https://oversea.cnki.net/index/) online database, with the aim to promote the further development and clinical application of THP.

## Chemical Characteristics and Plant Sources of Tetrahydropalmatine

**FIGURE 2:**
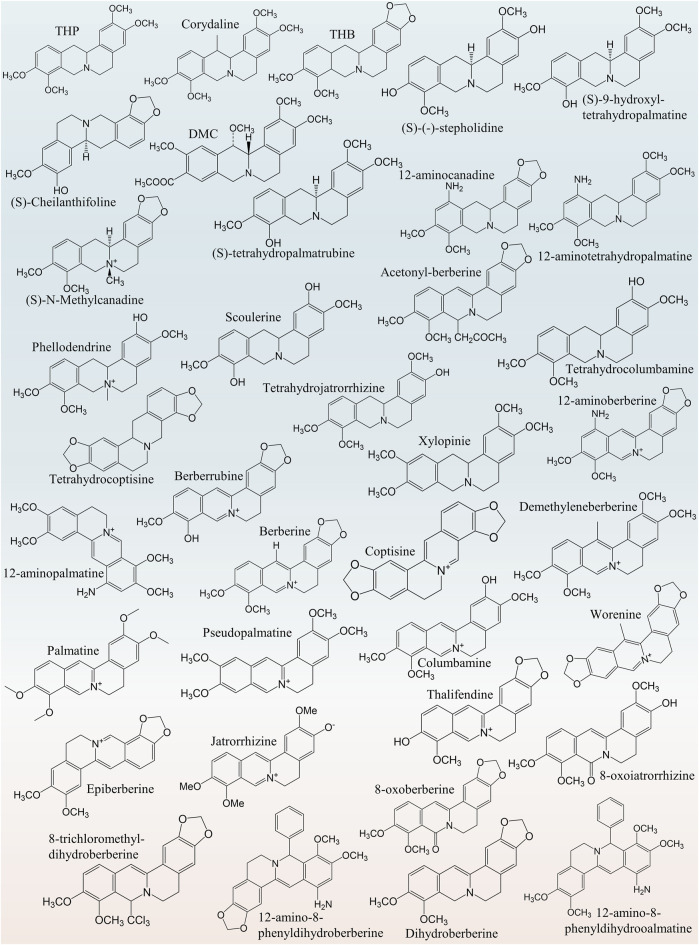
Chemical structural formulae of THP and its main related derivatives.

THP (molecular formula: C_21_H_25_NO_4_), an isoquinoline alkaloid, widely exists in Chinese herbal medicine preparations. THP has four–OCH_3_ groups at the 2, 3, 9 and 10 positions. THP has a chiral center in its structure, and its levorotatory, form [(-)-tetradropalmatine (-)-THP], is also known as rotundine. In L-THP, the N^+^ cation is downward, and the chiral (C_14_)-H is upward. Therefore, it is also known as (13aR)-5,8,13,13a-tetrahydro-2,3,9,10-tetramethoxy-6H-dibenzo [a,g]quinolizine hydrochloride (IUPAC name). THP has several derivatives with similar structures, such as corydaline (Figure 2), and benzyltetrahydropalmatine (Figure 2) (Jiang et al., 2009). Palmatine can be converted to THP when its C–C double bond and C–N double bond are reduced (Wei et al., 2001). Moreover, THP is a tertiary amine alkali and thus commonly soluble in trichloromethane, benzene, ether, hot ethanol (Li, 2013), and water (Kocanci and Aslim, 2021) and insoluble in other highly polar solvents (Li, 2013). The chemical structures of THP and its derivatives are presented in Figure 2. THP is a secondary metabolite generated during plant metabolism and found in peanuts, roots, tubers, leaves, or whole plants of some important medicinal plants (Supplementary Table S1), such as *Stephania cepharantha* Hayata [Menispermaceae; Shan Wu Gui] (Wu et al., 2011; Xiao et al., 2019), *Corydalis yanhusuo* (Y.H.Chou & Chun C.Hsu) W.T.Wang ex Z.Y.Su and C.Y.Wu [Papaveraceae; Corydalis rhizoma] (Xiao et al., 2011; Xu et al., 2015; Wu et al., 2018; Zhang et al., 2020), and *Uvaria microcarpa* Champ. ex Benth [Annonaceae; Zi Yu Pan] (Annonaceae) (Liu et al., 2011). In conclusion, by summarizing relevant literature on the plant sources of THP, we found that THP has a wide range of plant sources and provided a basis for the extraction and separation of THP.

## Pharmacological Activities of Tetrahydropalmatine

### Anti-Addiction Activity

The treatment of addiction is a complex problem. Many compounds derived from traditional Chinese medicine have been proved to have good anti-addiction activity. THP completely inhibits the effects of methamphetamine (METH) in all stages of the conditional place preference (CPP) task (Su et al., 2013). From the perspective of neuropharmacology, THP can selectively activates key regions of the brain’s dopaminergic, serotonergic, and norepinephrine systems according to pharmacological magnetic resonance imaging (Liu et al., 2012). Additionally, THP can significantly inhibit morphine-induced CPP acquisition and expression in a dose-dependent manner (Jiang et al., 2020). Treatment with THP blocks the morphine-induced downregulation of dopamine D2 receptors (D_2_R) and upregulation of GluA1 AMPA receptors in the prefrontal cortex, hippocampus (Hip), and striatum. THP inhibits dopamine-induced dopamine D1 receptor (D_1_R) activity and dopamine autoreceptor activity (Wu et al., 2018; Ahn et al., 2020). These results suggest that the possible mechanism may be linked to antagonizing morphine-induced changes in brain dopamine and glutamate transmission. Furthermore, the mechanism by which THP improves METH-induced behavioral phenotype is regulating brain-derived neurotrophic factor pathway, 5-HT neuronal activity, and dopamine D3 receptor (D_3_R) expression (Yun, 2014; Liu et al., 2021b). Meanwhile, THP combined with low-dose naltrexone (LDN) is used to treat cocaine relapse(Sushchyk et al., 2016). Combined treatment with THP and LDN can upregulate plasma β-endorphin and hypothalamic pro-opiomelanocortin. THP inhibits nicotine addiction by blocking neuronal α 4 β 2-nAChR functions and elevating extracellular dopamine levels in the nucleus accumbens shell, reducing nicotine intake and preventing relapse (Faison et al., 2016; Huang et al., 2021). The combination of L-THP and imperatorin (IMP) synergistically reduces EtOH-induced CPP, and the inhibition of inflammatory cytokines and the regulation of neurotransmitter receptor levels are the potential pharmacological mechanisms (Xu et al., 2021). Moreover, THP decreases ethanol drinking and the mechanism associated with D_2_R-mediated PKA signaling in the caudate-putamen (CPu) (Kim et al., 2013). These results fully prove that the combination of THP and other drugs, such as LDN and IMP, can significantly increase its anti-addiction effect.

Although THP by itself does not induce CPP or conditioned position aversion, THP significantly reduces the high expression of METH-induced extracellular signal-regulated kinase (ERK) phosphorylation (Su et al., 2020). In addition, THP (20 mg/kg) inhibits the enhanced phosphorylation of ERK and cAMP-responsive element-binding proteins in CPu, nucleus accumbens (NAc), and prefrontal cortex (PFC), and Hip (Du et al., 2017; Du et al., 2021). THP improves METH-induced hyper-locomotor activity, locomotor sensitization, and concomitant ERK1/2 activation in the NAc and CPu (Zhao et al., 2014) and may prevent addiction through antioxidant, anti-inflammatory, and anti-apoptotic mechanisms (Zhang Y. et al., 2018). Furthermore, THP reverses the impairment of spatial memory acquisition and retention (Cao et al., 2018), and the mechanism may be related to the expression of ERK1/2 in the PFC (Chen et al., 2012). METH self-administration was reduced when treated with THP at different doses (1.25, 2.50, and 5.00 mg/kg) (Gong et al., 2016). When THP was administered at 2.50 and 5.00 mg/kg, the METH-induced recovery of METH-seeking behavior was prevented. Interestingly, neither dose had an effect on locomotor activity (Yue et al., 2012; Cao et al., 2018). The effects of THP (3 mg/kg) on recovery may not be related to nonspecific motor impairment (Figueroa-Guzman et al., 2011). Collectively, these findings suggest that the anti-addiction effect of THP may be mainly related to the inhibition of METH in all stages of a CPP task, providing a basis for revealing the anti-addiction effect of THP.

### Analgesic Activity

**FIGURE 3:**
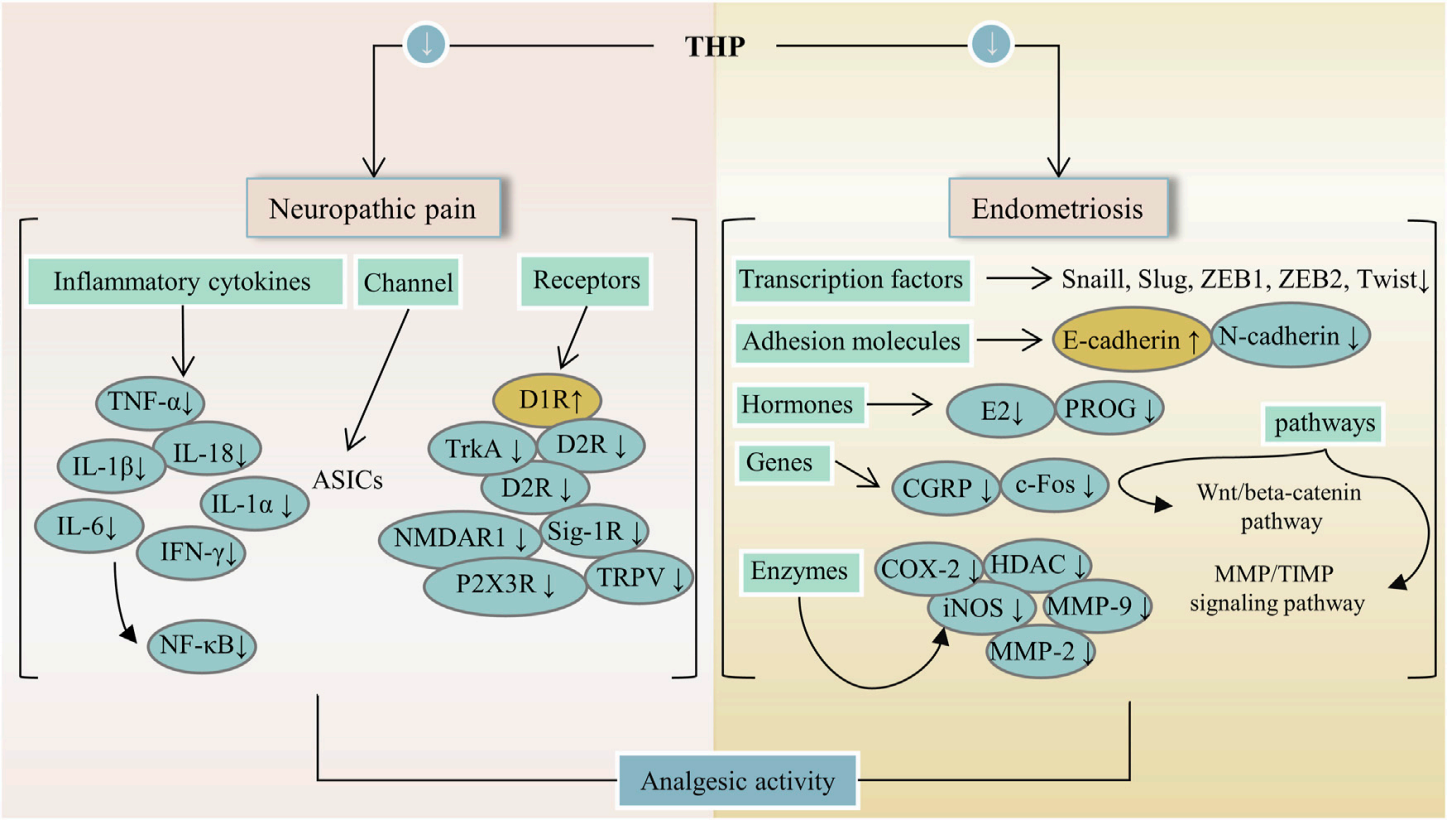
The main targets and pathways of analgesic activity of THP. Abbreviations:THP, Tetrahydropalmatine; TNF-α, Tumor necrosis factor alpha; IL-18, Interleukin-18; D1R, Dopamine D-1 receptor; D2R, Dopamine D-2 receptor; TrkA, Tropomyosin receptor kinase A; Sig-1R, Sigma-1 receptor; NMDAR1, N-methyl-D-asparate receptor 1; TRPV, Transient receptor potential vanilloid; P2X3R, P2X purinergic ion channel type 3 receptor; ZEB1, Zinc finger E-Box binding homeobox 1; ZEB2, Zinc finger E-Box binding homeobox 2; E2, Estrogen; PROG, Progesterone; CGRP, Calcitonin gene-related peptide; HDAC, Histone deacetylase; iNOS, Inducible NO synthase; MMP-2, Matrix metalloproteinase-2; MMP-9, Matrix metalloproteinase-9; MMP/TIMP, Matrix metalloproteinase/tissue inhibitor of MMP.

In China, some traditional Chinese medicines (e.g., *Corydalis yanhusuo* (Y.H.Chou & Chun C.Hsu) W.T.Wang ex Z.Y.Su and C.Y.Wu [Papaveraceae; Corydalis rhizoma]) containing THP are extensively used to treat pain. THP has an outstanding analgesic effect with different mechanisms and can decrease the abundance of protonated current mediated by acid-sensing ion channels (ASICs) in rat dorsal root ganglion (DRG) neurons, inhibit the functional activity of isolated primary sensory neuron ASICs, and relieve pain caused by acidosis (Liu et al., 2015). Mice that received an intraperitoneal injection at doses of 5 and 10 mg/kg showed increased mechanical threshold, thermal latency, and nonrapid eye movement sleep because THP had analgesic effects through D_1_R agonist and D_2_R antagonism (Liu YY. et al., 2019). Furthermore, THP can inhibit formalin-induced second-stage pain behavior through the sig-1R mechanism in the spinal cord (Kang et al., 2016). Moreover, THP can produce anti-hyperalgesia effects in a dose-dependent manner by intensifying dopaminergic transmission mediated by D_1_R (Zhou et al., 2016). In addition, the analgesic effect of THP on bone cancer pain induced by tumor cell implantation was observed in rats. In fact, THP can prevent or reverse bone cancer-related pain behavior because it inhibits microglial activation and proinflammatory cytokine increase (Zhang et al., 2015; Liu et al., 2021a). A paw withdrawal threshold test showed that THP had a dose-dependent analgesic effect on oxaliplatin-induced neuropathic pain in mice and possessed a strong analgesic effect on neuropathic pain in mice (Guo et al., 2014). A bee venom test was used in determining the antinociception of THP in rats. Accordingly, THP may be more effective for supraspinal processed nociceptive behavior than spinally mediated nociceptive behavior (Cao et al., 2011). Pain relief is a major goal of medications for endometriosis. One study conducted surgery on a rat to induce endometriosis. THP significantly reduced the size of the injury and significantly improved response to heat-damaging stimuli. The treatment expressively reduced immune reactivity to all mediators involved in central sensitization, such as histone deacetylase 2 (HDAC2) in dorsal root ganglion (DRG) and tyrosine kinase receptor A (TrkA) and calcitonin gene-related peptide (CGRP) in ectopic endometrium (Zhao et al., 2011). In addition, treatment with THP can inhibit myometrium infiltration, alleviate systemic hyperalgesia, and reduce uterine contraction amplitude and irregularity (Mao et al., 2011). THP can significantly reduce the severity of experimental primary dysmenorrhea. When THP is utilized with imperatorin (IMP), the severity of experimental primary dysmenorrhea was alleviated more effectively than THP or IMP alone. Mechanisms may include reduction of oxidative stress, inhibition of excessive inflammatory response, and reduction of *in vitro* rat uterine contraction by inhibition of extracellular Ca^2+^ influx (Chen et al., 2013). THP is a potent ingredient that alleviates dysmenorrhea in women. Moreover, a combination of ligustrazine, ferulic acid, and THP suppresses epithelial-mesenchymal transformation by inactivating MMP/TIMP signaling and Wnt/beta-catenin pathway in endometriosis (Chen et al., 2018; Tan et al., 2021; Zhang et al., 2021). In summary, the analgesic effects of THP mainly include relief of neuropathic pain and pain induced by endometriosis. Figure 3 lists the potential analgesic targets and pathways of THP. It may act through several pathways, including the MMP/TIMP signaling and Wnt/β-catenin pathways. Notably, THP can be used in the treatment of bone cancer pain. However, further research is required to determine whether it can be used to alleviate pain caused by other cancer types.

### Anti-Inflammatory Effects

**FIGURE 4:**
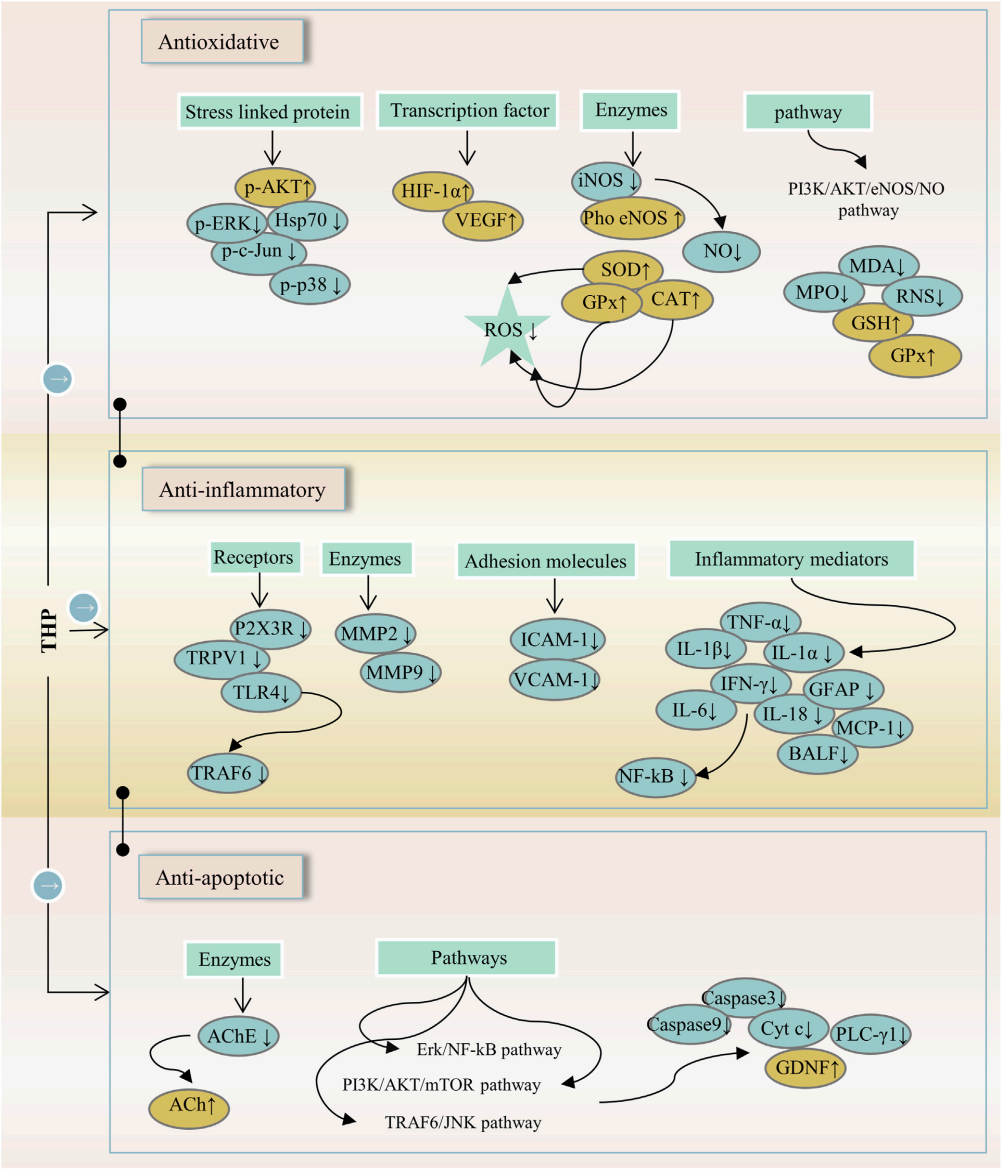
The main targets and pathways of anti-inflammatory activity of THP. Abbreviations: p-AKT, Phosphorylated AKT; p-ERK, Phosphorylated extracellular signal-regulated kinase; Hsp70, 70-kDa heat shock protein; p-c-Jun, Phosphorylated c-Jun; p-p38, Phosphorylated p38; HIF-1α, Hypoxia-inducible factor 1-alpha; VEGF, Vascular endothelial growth factor; iNOS, Inducible NO synthase; Pho eNOS, Phosphorylated endothelial nitric oxide synthase; NO, Nitric oxide; SOD, Superoxide dismutase; GPx, Glutathione peroxidase; CAT, Catalase; ROS, Reactive oxygen species; PI3K/AKT/eNOS/NO, Phosphatidylinositol 3-kinase/AKT/endothelial nitric oxide synthase/nitric oxide; MDA, Malondialdehyde; MPO, Myeloperoxidase; RNS, Reactive nitrogen species; GSH, Glutathione; P2X3R, P2X purinergic ion channel type 3 receptor; TRPV1, Transient receptor potential vanilloid 1; TLR4, Toll-like receptor 4; TRAF6, TNF-receptor associated factor-6; MMP2, Matrix metalloproteinase-2; MMP9, Matrix metalloproteinase-9; ICAM-1, Intercellular adhesion molecule-1; VCAM-1, Vascular cell adhesion molecule-1; TNF-α, Tumornecrosis factor alpha; IL-1β, Interleukin-1 beta; IL-1α, Interleukin-1 alpha; IFN-γ, Interferon-gamma; GFAP, Glial fibrillary acidic protein; IL-6, Interleukin-6; IL-18, Interleukin-18; MCP-1, Monocyte chemoattractant protein-1; BALF, Bronchoalveolar lavage fluid; NF-κB, Nuclear factor-kappa B; AChE, Acetylcholinesterase; ACh, Acetylcholine; Erk/NF-κB, Extracellular signal-regulated kinase/nuclear factor-kappa B; PI3K/AKT/mTOR, Phosphatidylinositol 3-kinase/Akt/mechanistic target of rapamycin; TRAF6/JNK, TNF-receptor associated factor-6/c-Jun N-terminal kinase; Cyt c, Cytochrome c; PLC-γ1, Phospholipase C gamma 1; GDNF, Glial cell-derived neurotrophic factor.

Inflammation is involved in the progression and development of many clinical diseases, such as atherosclerosis, pneumonia, and hepatitis. Hence, anti-inflammatory drugs remain a major topic of interest. THP can alleviate neuropathic and inflammatory pain by downregulating P2X ligand-gated ion channel 3 (P2X3) receptors and transient receptor potential vanilloid 1 (TRPV1), which play a key role in the occurrence and maintenance of pain (Wang et al., 2021). The anti-inflammatory effect of THP on acute lung injury (ALI) induced by limb-ischemia/reperfusion (I/R) surgery was found *in vivo* (Wen et al., 2020). The phosphatidylinositol 3-kinase (PI3K)/protein kinase B (AKT)/mechanistic target of rapamycin (mTOR) is crucial to the regulation of cellular growth and metabolism. THP protects ALI, and possible mechanisms are associated with the inhibition of PI3K/Akt/mTOR phosphorylation (Zhang et al., 2015). THP can inhibit ERK/nuclear factor κB (NF-κB) signaling pathway and reduce hepatocyte apoptosis and autophagy (Yu et al., 2019). In RAW264.7 macrophages, THP decreases the expression of various proinflammatory cytokines (TNF-α, IL-1α, and IL-1β) in a dose-dependent manner possibly because of the inhibition of the NF-κB signaling pathway (Yodkeeree et al., 2018; Zhi L. et al., 2020). In a myocardial IR injury model, THP reduces inflammatory cytokines (TNF-a and MPO), which are associated with PI3K/Akt/eNOS/NO pathway activation (Han et al., 2012). In a mouse tumor-cell-implantation-induced pain model, THP decreases the levels of TNF-α and IL-18 but had no effect on IL-1β (Zhang et al., 2015). Moreover, THP reduces the release of inflammatory cytokines (IL-6 and TNF-α) and inhibits apoptosis and autophagy through the TRAF6/JNK pathway (Yu et al., 2018). Additionally, THP down-regulates the transcription and translation levels of vascular cell adhesion molecule-1 and the mRNA and protein levels of TNF receptor-associated factor-6, Toll-like receptor 4, and intercellular adhesion molecule-1 (Yang et al., 2015; Sun C. et al., 2018). In a mouse Japanese encephalitis virus (JEV) model, THP inhibited a decrease in proinflammatory cytokines (THF-α, MCP-1, IFN-γ, and IL-6) (Lixia et al., 2018). THP exerts a potential radio-protective effect on irradiation-induced lung injury (Yu et al., 2016). Given that THP not only reduces bronchoalveolar lavage fluid (BALF) cell recruitment but also reduces BALF protein levels. THP decreases the expression of monocyte chemotactic protein-1, NF-κB, and glial fibrillary acidic proteins (Qu et al., 2016; Wang et al., 2018). These results suggest that the anti-inflammatory effect of THP is closely related to the P2X3/TRPV1, TRAF6/JNK, PI3K/Akt/eNOS/NO, NF-κB, and ERK/NF-κB signaling pathways (Figure 4).

### Neuroprotective Activity

**FIGURE 5:**
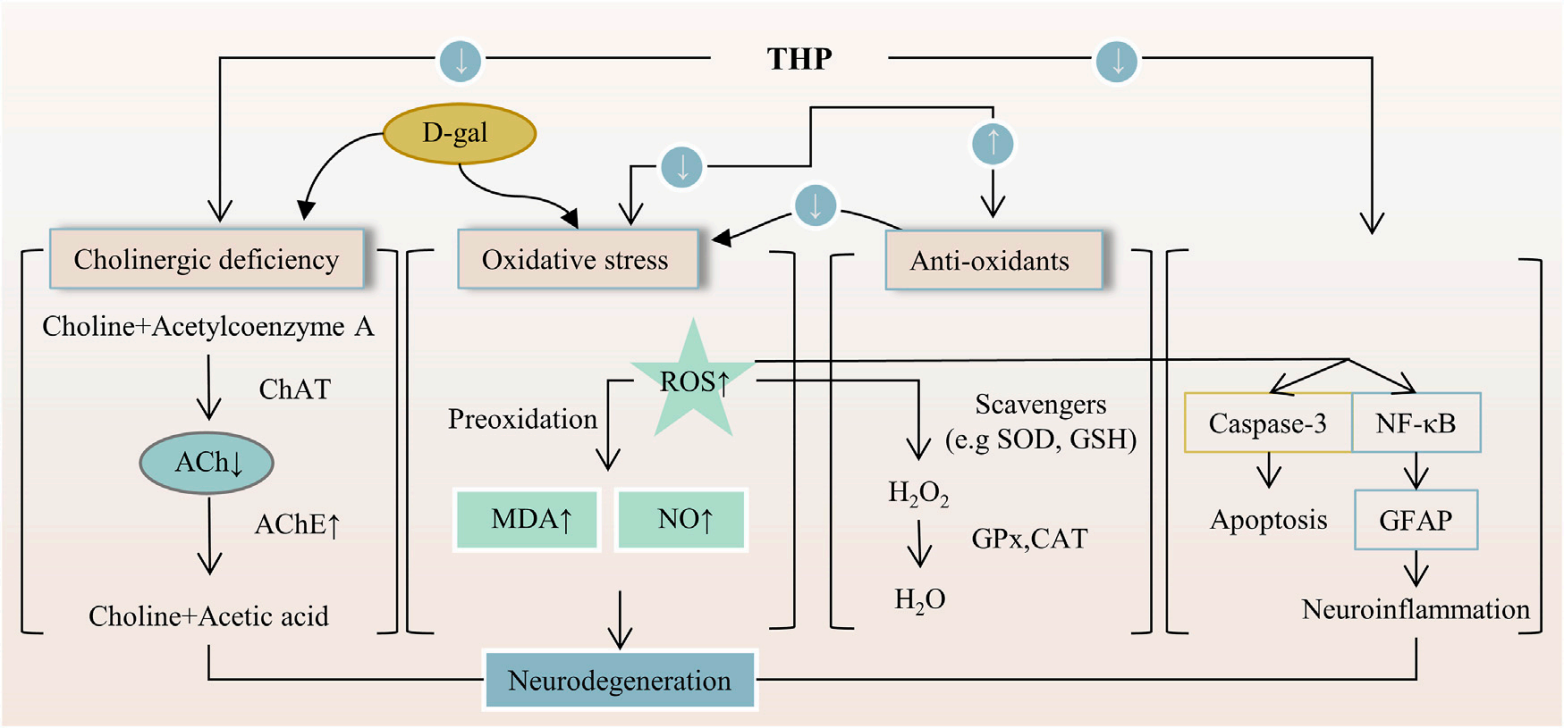
The main targets and pathways of neuroprotective activity of THP. Abbreviations:D-gal, D-galactose; ChAT, Choline acetyltransferase; ACh, Acetylcholine; AChE, Acetylcholinesterase; ROS, Reactive oxygen species; MDA, Malondialdehyde; NO, Nitric oxide; SOD, Superoxide dismutase; GSH, Glutathione; GPx, Glutathione peroxidase; CAT, Catalase; NF-κB, Nuclear factor-kappa B; GFAP, Glial fibrillary acidic protein.

The effect of neuroprotective agents is to reduce the cell damage after ischemia, so as to prolong the time window of cerebral perfusion therapy and achieve the purpose of delaying the death of nerve cells and alleviating brain dysfunction. THP has a neuroprotective effect on neuronal apoptosis induced by brain I/R injury (Sun R. et al., 2018). THP can improve c-Abl expression and neuronal apoptosis. The number of viral populations; expression level of caspase-2; levels of reactive oxygen species, nitrogen, microglia, proinflammatory mediators, and stress-related protein molecules; and neuronal apoptosis decreased after the THP treatment of JEV (Lixia et al., 2018). THP can inhibit the delayed rectifier Kv1.5 channel expressed in HEK293 cells (HEK293 is a cell line derived from human embryonic kidney cells grown in tissue culture). This line was initiated by the transformation and culturing of normal HEK cells with sheared adenovirus 5 DNA (Li K. et al., 2017). The antinociceptive effect of THP is related to the modulation of spinal sigma-1 receptor (Sig-1R) activation (Kang et al., 2016). On D-galactose-induced memory impairment in rats, THP not only reverses the abnormal levels of acetylcholine and acetylcholinesterase activities related to several neuropsychiatric functions, such as learning, memory, and sleep. But also reduces the expression of NF-κB and glial fibrillary acidic protein (GFAP) (Qu et al., 2016). Activated NF-κB can increase the expression of inflammatory cytokines and astrocytes, thus causing memory impairment. Meanwhile, GFAP is a specific marker of astrocyte activation (Ali et al., 2015). Likewise, THP inhibits the functional activity of ASICs. In addition, THP can alter the membrane excitability of rat DRG neurons to acid stimulation and significantly reduce action potential and depolarization amplitude induced by extracellular pH drop (Liu et al., 2015), as shown in Figure 5. THP has potential anxiolytic-like and antidepressant effects and can inhibit a decrease in hypothalamic neuropeptide Y level. This effect is related to a predisposition to anxiety or stress-induced depression (Serova et al., 2013). THP can inhibit the increase in the expression level of the adrenocorticotropin-releasing factor in the hypothalamus. Important genes involved in serotonin, dopamine, acetylcholine, and gamma-aminobutyric acid neurotransmitter systems showed significant transcriptional folding changes in rodent models of post-traumatic stress disorder after the subcutaneous injection of THP (Ceremuga et al., 2013; Lee B. et al., 2014). Overall, these results suggest that THP has a good neuroprotective effect, including anti-memory damage, antidepression, and anti-anxiety effects. These neuroprotective effects may be realized through targets and pathways, such as inhibiting neuronal apoptosis, reducing the level of free radicals, regulating inflammatory factors and their pathways, and regulating neurotransmitters and related receptors.

### Anticancer Activity

Cancer is a serious threat to human health and quality of life and has high morbidity and mortality. To date, no miracle drug for cancer is available, and novel drugs for cancer treatment should be developed. A recent study confirmed that THP can treat glioblastoma multiforme by inhibiting the ERK/NF-κB cascade (Xue and Chen, 2021). THP is effective in treating melanoma by inhibiting the activity of CDK2, which is a unique target among the CDK family members in melanoma therapy (Tang and Chen, 2014). In ovarian cancer A2780/DDP cell line, THP can increase the sensitivity of ovarian cancer cells to cisplatin by regulating the miR-93/PTEN/AKT pathway (Gong et al., 2019). Moreover, THP alleviates kidney injury induced by cisplatin through the selective inhibition of organic cation transporter 2 (OCT2) but does not affect its antitumor effect (Li et al., 2020). THP can inhibit the uptake of oxaliplatin (OXA), which has severe peripheral neurotoxicity (Yi et al., 2021). In another report, p53 null leukemia EU-4 cells coped with THP, and the result showed that THP downregulated XIAP protein by inhibiting MDM2, which is a primary cellular inhibitor of p53 and a therapy target of cancer (Wang S. et al., 2017) and is associated with proteasome-dependent pathway; hence, THP result in p53-independent apoptosis and increased the sensitivity of EU-4 cells to doxorubicin (Li S. et al., 2017). In addition, THP increases the sensitivity of ER alpha (+) BCa cells to tamoxifen and fulvestrant, which are the inhibitors of antiestrogen and are applied to patients with ERα-positive breast cancer (Xia et al., 2020). *In vitro*, THP markedly restrains the proliferation of ER alpha (+) BCa cells by inducing cell cycle arrest rather than apoptosis. Nitidine chloride has anticancer activity. As an OCT2 inhibitor, THP can reduce its accumulation and toxicity in the kidney, and as OCT1 and OCT3 inhibitors, THP can reduce their accumulation and toxicity in the liver (Li et al., 2014; Li et al., 2016). These results suggest that THP can be used as a potential treatment compound for glioblastoma multiforme, melanoma, ovarian cancer, leukemia, and breast cancer and can attenuate cancer patients’ resistance to anticancer drugs, such as cisplatin, doxorubicin, fulvestrant, and tamoxifen. However, the underlying mechanism of THP in combination with other chemicals has not been fully elucidated and will be the focus of future research.

### Other Pharmacological Activities

**FIGURE 6:**
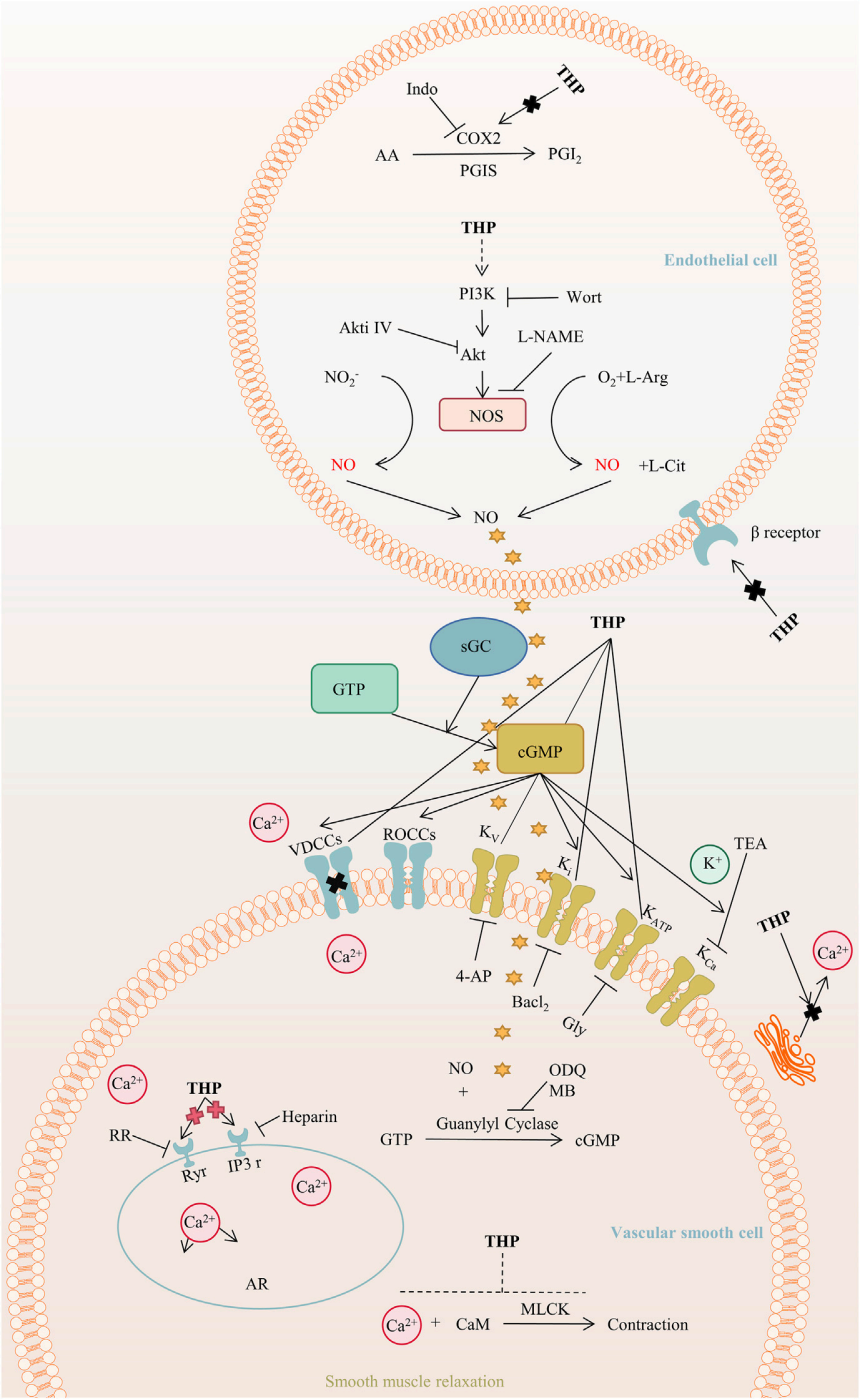
The main targets and pathways of vasodilatory activity of THP. Abbreviations:THP, Tetrahydropalmatine; Indo, Indomethacin; COX2, Cyclooxygenase-2; PGI_2_, Prostacyclin; PGIS, Prostacyclin synthase; AA, Arachidonic acid; PI3K, Phosphatidylinositol 3-kinase; Wort, Wortmannin; Akt, Protein kinase B; Akti Ⅳ, Akt inhibitor Ⅳ; L-NAME, L-nitroarginiemethylester; NOS, Nitric oxide synthase; L-Arg, L-arginine; NO, Nitric oxide; L-Cit, L-Citrulline; GTP, Guanosine triphosphate; sGC, Soluble guanylyl cyclase; cGMP, Cyclic guanosine monophosphate; VDCCs, Voltage-dependent calcium channels; ROCCs, Receptor-operated calcium channels; K_v_, Voltage-dependent potassium channel; K_i_, Inward rectifying potassium channel; K_ATP_, ATP-sensitive potassium channel; K_Ca_, Calcium-activated potassium channel; TEA, Tetraethylammonium; 4-AP, 4-Aminopyridine; Gly, Glibenclamide; ODQ, 1H-[1,2,4]-oxadiazolo-[4,3-alpha]-quinoxalin-1-one; MB, Methylene blue; RR, Ruthenium red; Ryr, Ryanodine receptors; IP3r, IP3 receptors; AR, Aorta relaxation; CaM, Calmodulin; MLCK, Myosin light chain kinase.

THP exerts hypotensive effects. The post-perfusion of THP (15 mg/kg/day) can reduce systolic blood pressure by decreasing diameter (Wang et al., 2018). The mechanism of the THP dilation of the rat aorta mainly involves the PI3K/Akt/eNOS/NO/cGMP signaling pathway and Ca^2+^ and K^+^ channels but not COX2, β-adrenergic receptor, and renin-angiotensin system (Zhou et al., 2019). Further, the activation of NO/cGMP signaling pathways results in the activation of an endothelium-dependent pathway (Qu et al., 2015). In addition, THP activates the K_ATP_ channel and plays a role in vascular relaxation, and promotes angiogenesis by regulating the sequence of citrulline-to-arginine flux, arginine biosynthesis, and VEGFR2 expression in endothelial cells (Cui et al., 2021), as shown in Figure 6. Furthermore, THP can inhibit osteoclast formation in a dose-dependent manner and has no cytotoxicity at a concentration lower than 19.00 μg/ml (Zhi X. et al., 2020). In *vitro* experiments, THP suppressed early osteoclast differentiation, downregulated the transcription level of osteoclast-related genes, and impaired the function of osteoclasts in bone marrow monocytes cells and mouse leukemic monocyte/macrophage cell line RAW264.7 cells. *In vivo*, THP significantly inhibits ovariectomy-induced bone loss and osteoclast formation in mice. It mineralizes nodule density and increases osteoblast proliferation (Wang et al., 2019).

**FIGURE 7:**
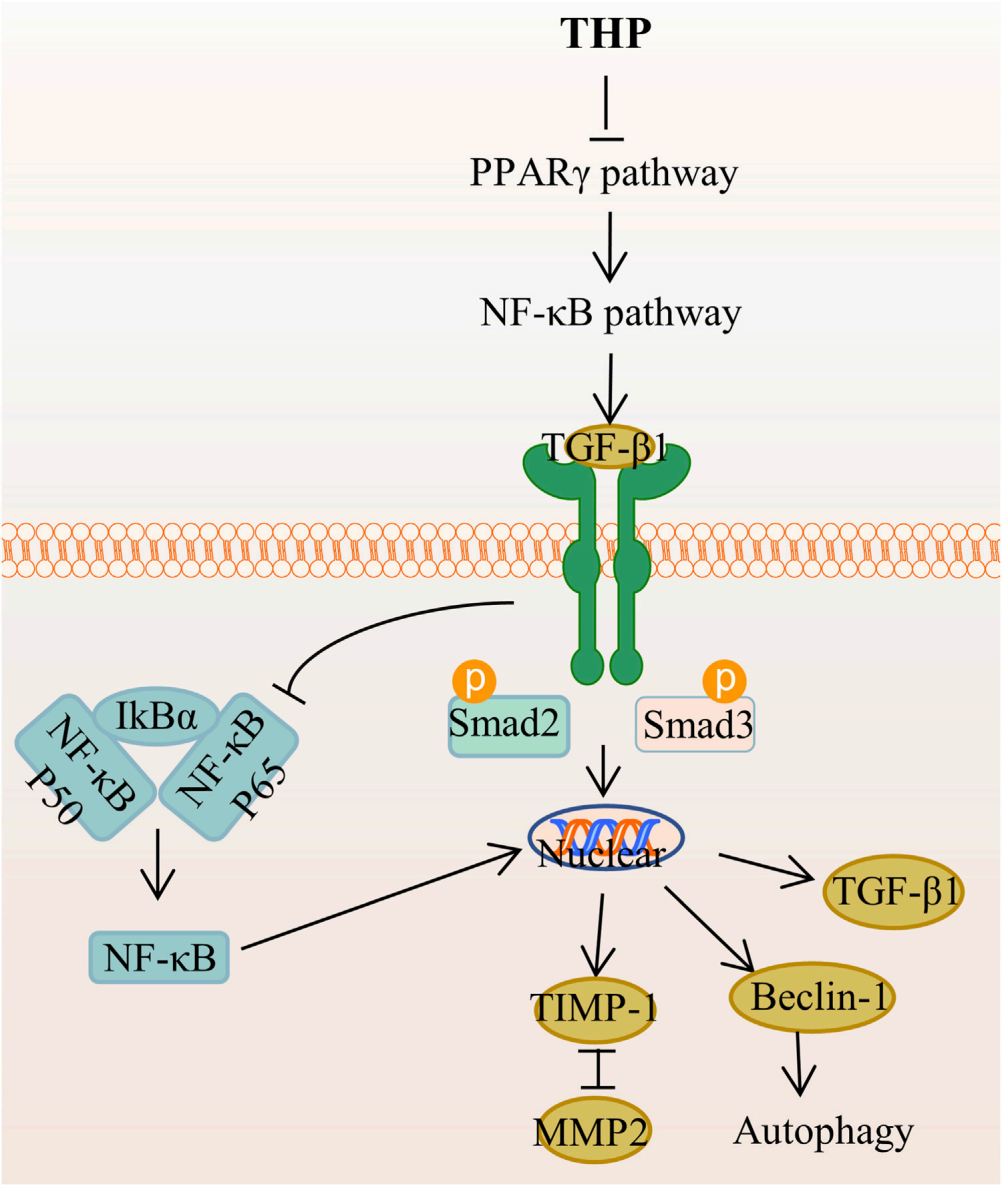
The main targets and pathways of anti-hepatic fibrosis activity of THP. Abbreviations:THP, Tetrahydropalmatine; PPARγ, Peroxisome proliferator-activated receptor gamma; NF-κB, Nuclear factor-kappa B; TGF-β1, Transforming growth factor-β1; IκBα, Inhibitor of kappa B alpha; Smad2, Drosophila mothers against decapentaplegic protein 2; Smad3, Drosophila mothers against decapentaplegic protein 3; TIMP-1, Tissue inhibitor of metalloproteinase 1; MMP2, Matrix metalloproteinase 2.

THP can enhance MyoD activation through the upregulation of p38MAPK and Akt, which can promote premature muscle development (Lee SJ. et al., 2014). Accordingly, THP can serve as a potential drug for preventing fibrosis and promoting muscle regeneration and repair. The mechanism of THP in anti-adipogenic effect is that THP suppresses hepatic lipid accumulation and decreases the serum levels of serum cholesterol, triglyceride, low-density lipoprotein cholesterol, and high-density lipoprotein cholesterol, as demonstrated in golden hamsters fed with a high-fat diet (Sun C. et al., 2018). In addition, THP inhibits lipid accumulation and decreases the level or activity of lipid droplets, triglyceride, and glycerol-3-phosphate dehydrogenase in 3T3-L1 adipocytes through the AMPK signaling pathway (Piao et al., 2017). THP inhibits extracellular matrix (ECM) deposition and hepatic stellate cells (HSCs) autophagy by regulating the PPAR gamma/NF-κB and TGF-beta 1/Smad pathways, thereby reducing liver fibrosis, which is a necessary stage in the progression of chronic liver disease to cirrhosis (Yu et al., 2021) (Figure 7). Other studies demonstrated that THP has a certain resistance to plasmodium(Baghdikian et al., 2013; Bory et al., 2013; Malebo et al., 2013), parasites (Dutta et al., 2016), and pathogenic fungi (Zhao et al., 2019).

## Pharmacokinetic Characteristics

With the advances in pharmaceutical chemistry, the demand for pharmacokinetic characteristics of drugs is increasing. At present, the properties and application prospects of patent drugs are not only evaluated by their effectiveness and low toxicity but also by their good pharmacokinetic characteristics (Yang et al., 2021). The metabolic pathways of THP in three male healthy Chinese volunteers were studied (Xiao et al., 2016). The biotransformation of THP mainly includes monohydroxylation, demethylation, glucuronidation, and sulfonation of demethylated metabolites. Multiple demethylations, glucuronic acid and sulfate coupling, and renal excretion of THP in humans are the main drug clearance pathways of THP. THP has four monodesmethyl metabolites: L-isocorypalmine, L-corypalmine, L-tetrahydropalmatrubine, and L-corydalmine (Abdallah et al., 2017). The mean time to peak concentration (T_max_) and half-life (T_1/2_) of THP in rats were 0.44 and 4.49 h, respectively (Wang W. et al., 2017), whereas those of healthy cocaine-using adults were 1.5 and 13.3 h, respectively (Hassan et al., 2017). THP is only partially converted into metabolites, and the prototype drug remains in the blood (Wang W. et al., 2017). THP and its metabolites show high blood-brain barrier (BBB) permeability because of their high lipophilic properties (Wang et al., 2012; Abdallah et al., 2017). From the pathological perspective, the T_1/2_ and MRT of THP in spontaneously hypertensive rats (SHR) were significantly longer than those in healthy Sprague-Dawley rats, indicating that the elimination of THP in SHR is slow (Hong et al., 2012). THP can be metabolized by the rat gut microbiota, indicating the importance of intestinal microorganisms in THP metabolism (He et al., 2017). THP can be transported to the kidney through OAT3, OATP1B1, and OATP1B3 and then to the liver. The ingredients in *Angelica dahurica* (Fisch. ex Hoffm.) Benth. et Hook. f. ex Franch. et Sav. inhibited both pathways and increased THP levels in the blood and brain (Wang et al., 2020). Other components in Yuanhu Zhitong prescription (Zhang H. et al., 2018) or Tong-Bi-Si-Wei-Fang (Ni et al., 2019) can increase the Cmax of THP or prolong the retention time of THP in the plasma. THP enantiomers in human liver microsomes are mainly metabolized by CYP3A4/5 and CYP1A2, and (+)-THP is preferentially metabolized by CYP1A2. CYP3A4/5 has the same contribution to (-)-THP or (+)-THP metabolism (Sun et al., 2013). In rat liver microsomes, THP enantiomers are mainly metabolized by CYP3A1/2 and CYP1A2, and CYP3A1/2 tends to metabolize (+)-THP, whereas CYP1A2 tends to metabolize (-)-THP (Zhao M. et al., 2012). d-THP inhibits the isozyme activities of CYP2D6 and CYP1A2, whereas L-THP inhibits the isozyme activities of CYP1A2 and induces the isozyme activities of CYP3A4 and CYP2C9 (Zhao Y. et al., 2012; Li et al., 2015). The above performance of THP is related to the connection of the H-bond and a few Pi-bond with CYP1A2-, CYP2D6-, and CYP3A4-specific amino acid residues (Zhao et al., 2015). THP enantiomers inhibit P-gp but not MRP1 or BCRP, and (-)-THP and (+)-THP show the obvious stereoselective difference (Sun et al., 2012). By contrast, CYP inhibitors significantly affect the systemic levels of THP and its metabolites (Xiao et al., 2021). Moreover, the effective oral dose of DA-9701, which is a new botanical gastroprokinetic agent, can decrease the brain concentrations of THP and inhibit THP from exerting central D_2_R antagonism (Jung et al., 2015).

Traditional oral administration may not be the best use of THP because of poor intestinal absorption, rapid clearance, and low bioavailability. To improve absorption efficiency, the absorption level and rate of THP in a self-microemulsifying drug delivery system (SMEDDS) pellet formulation are higher than those of raw excipients(Tran et al., 2016). The SMEDDS improved the oral bioavailability of THP in a rabbit model by 198.63% (Tung et al., 2018) and in a rat model by 225% (Li et al., 2021) compared with THP suspension. The figures for self-emulsifying drug-delivery systems were 33.2% in a rat model (Ma et al., 2012) and 234.77% for binary amorphous solid dispersion application in rabbit plasma (Tung et al., 2021). Another method for increasing THP bioavailability is the use of hydrochloride freeze-dried powder, in which C-max, AUC, and bioavailability are significantly elevated (Wu C. et al., 2013). THP oral disintegrating tablets have good taste and tolerance, rapidly disinteg, and are quickly absorbed (Chao-Wu et al., 2011). A comparison of plasma pharmacokinetics and lung distribution of THP at the Feishu point (BL 13) and non-Feishu points showed that the amount of THP entering the blood and lung after Feishu point application was significantly higher than that after non-Feishu point application (Lin et al., 2014). Moreover, the *in vitro* release of THP with the addition of osmotic promoters showed an abnormalrate (non-Fickian) release kinetics (Li et al., 2011).

### Toxicological Characteristics of Tetrahydropalmatine

**FIGURE 8:**
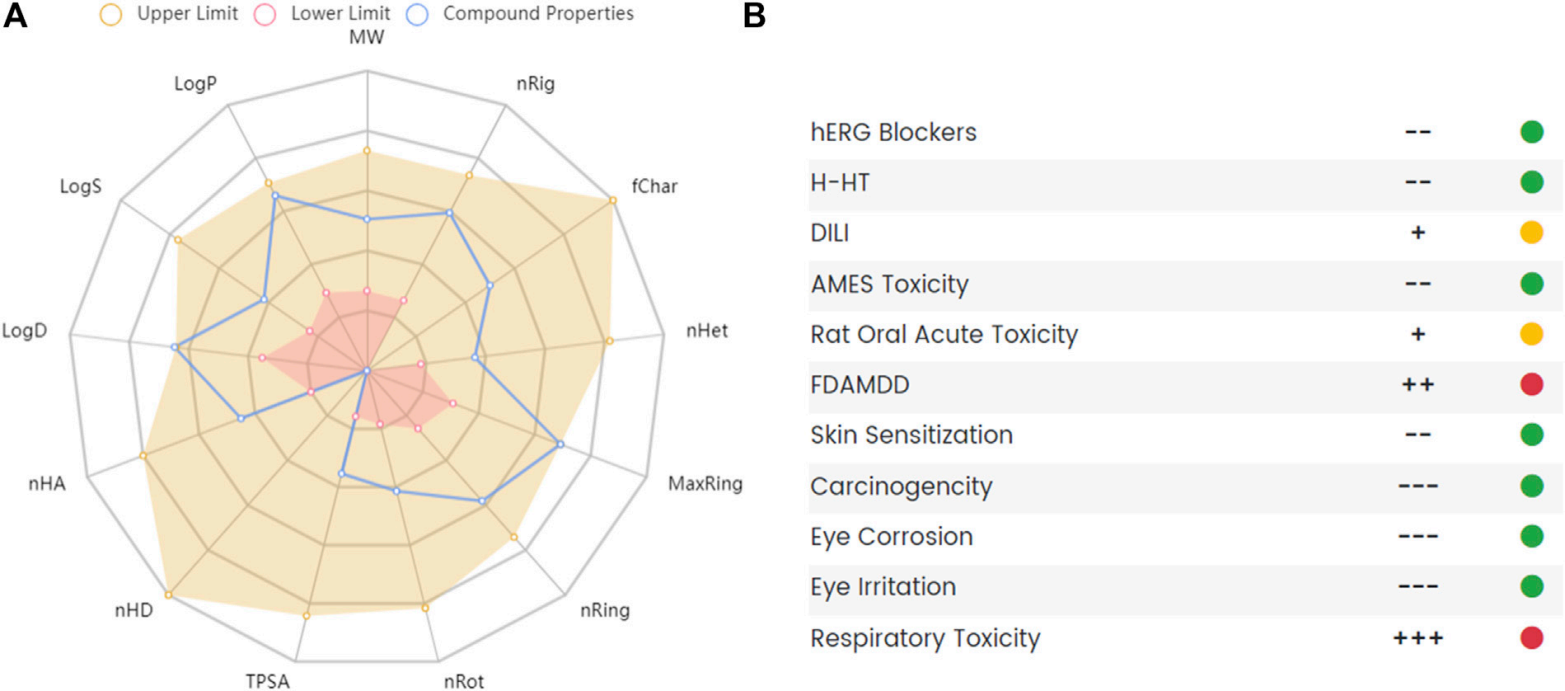
The absorption, distribution, metabolism, excretion and toxicity (ADMET) of THP. **(A)** Radar map of THP ADMET. **(B)** The property and decision of THP toxicity. The data obtained from the following websites: https://admetmesh.scbdd.com/.

Studies used THP or corydalis with caution in patients with heart diseases because of its potential cardiac and neurotoxic effects (Chan et al., 1999). A randomized, placebo-controlled, and double-blind clinical study assessed the safety of THP for cocaine users. The results showed that a short 3.5-days course of THP was well tolerated and safe and did not affect the pharmacokinetics of cocaine or its acute cardiovascular effects (Hassan et al., 2017). The liver toxicity of THP in mice revealed that THP suppressed the expression of CYP1A2, and no obvious pathological changes were observed in liver tissues after THP administration (Wang D. et al., 2017). Some reports mentioned the toxicity of THP as a natural substance but did not study it in depth(Wu H. et al., 2013; Zeng et al., 2015). Although THP has proven to be a promising compound with a variety of pharmacological actions, it also has some disadvantages, such as poor intestinal absorption, rapid clearance, and some potential toxicity. To further clarify its pharmacokinetic and toxicological characteristics, we used ADMETlab 2.0 (Xiong et al., 2021) to predict the ADMET of THP. The ADMET characteristics of THP are shown in Figure 8. The results indicated that THP is toxic at concentrations above the maximum recommended daily dose by the FDA and potentially toxic to the respiratory system. In conclusion, experimental evaluation of THP toxicity is limited, especially in terms of the cytotoxicity, long-term toxicity, and acute toxicity of THP.

## Conclusion and Future Perspectives

In this review, the chemical properties, plant origins (Supplementary Table S1), pharmacological activities (Supplementary Table S2), and pharmacokinetic and toxicological characteristics of THP were systematically reviewed. The structure of THP has long been elucidated. THP comes from numerous natural plant sources and is mostly found in China and Southeast Asian countries. The extraction process needs to be strengthened. THP has a large number of pharmacological effects, including analgesic, anti-addiction, anti-inflammatory, neuroprotection, and anticancer effects. As a traditional gynecological analgesic, this aspect of pharmacological research is sufficient. There are also many studies on the anti-addiction and neuroprotective effects associated with the neuropharmacological effects of analgesia. Other important topics, such as anti-inflammatory and antitumor mechanisms, have been discussed. Further pharmacological effects of THP, such as antifibrosis, antiparasite, antifungal, and antimalaria, need further research. In addition, we also found that in addition to being used alone, THP can also be used in combination with other compounds to achieve the effect of increasing efficacy and reducing toxicity (Chen et al., 2013; Chen et al., 2018; Gong et al., 2019; Xu et al., 2021; Zhang et al., 2021). This also provides more evidence for THP combination.

**FIGURE 9:**
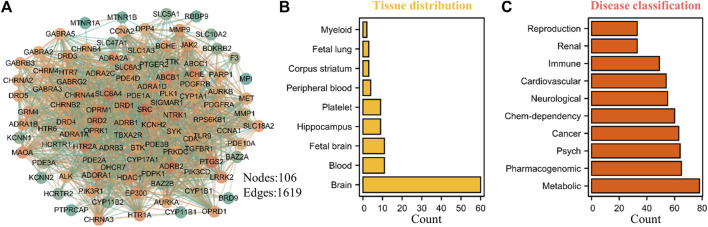
Potential target prediction, tissue distribution and disease type enrichment analysis of THP. **(A)** A total of 106 targets were predicted by SwissTargetPrediction database (http://www.swisstargetprediction.ch/). **(B–C)** The tissue distribution and disease type enrichment analysis of THP using DAVID database. The data obtained from the following websites: https://david.ncifcrf.gov/home.jsp.

The rapid development of bioinformatics and network pharmacology provides a powerful means for further exploring the potential potential pharmacological value of THP. Therefore, we predicted the potential targets of THP by using the SwissTargetPrediction database (Daina et al., 2019) and conducted target tissue location and clinical disease enrichment analysis of 106 potential targets (Figure 9A) with the DAVID database (Huang da et al., 2009). The results showed that nine items were enriched to tissue distribution: brain, blood, fetal brain, Hip, platelet, peripheral blood, corpus striatum, fetal lung, and myeloid (Figure 9B, Supplementary Table S3). Ten disease types had a Count% of ≥30: metabolic, pharmacogenomic, psych, cancer, chem-dependency, neurological, cardiovascular, immune, renal, and reproduction (Figure 9C, Supplementary Table S4). Further analysis showed that these potential targets were mostly abundant in brain tissues, metabolic diseases, nervous system, and other disease types, and these disease types are also the focus of our future research. In addition, whether DRD1, SRC, SLC6A4, NTRK1, DRD2, PTGS2, ADRB2 and other potential targets of THP play a role in these diseases. This also requires further experimental verification.

The drug absorption and metabolic pathways of THP have been described in animal models, but the pharmacokinetic studies of THP in humans are limited. Many drugs in traditional Chinese medicine prescriptions can increase the utilization rate of THP, and the application of THP in new preparations and dosage forms can improve its pharmacokinetic properties. Drug safety is an important factor affecting the therapeutic potential of THP. Although no obvious toxicity of THP has been observed to date, through literature review and analysis, we found that THP may have some potential toxicity. Given that toxicity tests on THP are still lacking, the potential toxicity of THP should be extensively explored, and studies in this field should be strengthened. In summary, this paper comprehensively reviewed and summarized the chemical properties, plant origins, pharmacological activities, pharmacokinetic and toxicological characteristics of THP, which provides a reference for future research and development of THP.
