# Supplementary material for: A Comprehensive Review on the Chemical Properties, Plant Sources, Pharmacological Activities, Pharmacokinetic and Toxicological Characteristics of Tetrahydropalmatine
Source: Front Pharmacol. 2022 Apr 26;13:890078. doi: 10.3389/fphar.2022.890078 (PMC9086320; doi:10.3389/fphar.2022.890078)
Supplement: Supplementary file 1 [file DataSheet1.docx]

**Supplementary materials**

**For**

**A Comprehensive Review on the Chemical Properties, Plant Sources, Pharmacological Activities, Pharmacokinetic and Toxicological Characteristics of Tetrahydropalmatine**

*Qinyun Du^1^, Xianli Meng^1,2*^, Shaohui Wang^3*^*

*^1^State Key Laboratory of Southwestern Chinese Medicine Resources, School of Pharmacy, Chengdu University of Traditional Chinese Medicine, Chengdu, of Traditional Chinese Medicine, Chengdu, China**, ^2^State Key Laboratory of Southwestern Chinese Medicine Resources, Innovative Institute of Chinese Medicine and Pharmacy, Chengdu University of Traditional Chinese Medicine, Chengdu, China, ^3^**State Key Laboratory of Southwestern Chinese Medicine Resources, School of Ethnic Medicine, Chengdu University of Traditional Chinese Medicine, Chengdu, China*

**Corresponding authors:** Shaohui Wang (winter9091@163.com), Xianli Meng (xlm999@cdutcm.edu.cn

**Table S1** The plant sources of tetrahydropalmatine.

| **Plant species** | **Effect** | **Area** | **Family** | **Used part** | **Extract** | **References** |
| --- | --- | --- | --- | --- | --- | --- |
| *Stephania epigaea* H.S.Lo | N/A | The south of China{Xiao, 2021 #3}, especially in Yunnan and Guangxi province | Menispermaceae | Tuber | Ethanol and ammonium sulfate | (Sun et al., 2020; Xiao et al., 2021) |
| *Stephania venosa* (Blume) Spreng. | Treatment of cancer and diabetes, and as a blood-tonic and aphrodisiac | Southeast Asian countries | Menispermaceae | Root/Tuber | Methanol | (Kongkiatpaiboon et al., 2016; Le et al., 2017) |
| *Stephania cephalantha* Hayata | Used to treat lung cancer | China | Menispermaceae | Earthnut | 60% ethanol | (Wu et al., 2011; Xiao et al., 2019) |
| *Stephania cambodica* Gagnep. | Used to treat anxiety, malaria, fever, wounds, joint pains, fatigue and male sexual dysfunction | Cambodia and Vietnam | Menispermaceae | Tuber | hydroethanolic extract/52% ethanol | (Dary et al., 2017b) (Dary et al., 2017a) |
| *Stephania rotunda* Lour. | Used to treat asthma, headache, fever, and diarrhoea | Southeast Asia, Cambodian, Laos, Indian and Vietnamese | Menispermaceae | Root, Stem and Tuber | dichloromethane  dichloromethane and aqueous extracts | (Baghdikian et al., 2013; Bory et al., 2013; Desgrouas et al., 2014) |
| *Stephania bancroftii* F.M.Bailey | N/A | N/A | Menispermaceae | Rhizome | Methanol | (Bartley et al., 1994) |
| *Stephania yunnanensis* H.S.Lo | N/A | Yunnan province | Menispermaceae | Tuber | N/A | (Ma et al., 2008) |
| *Stephania glabra* (Roxb.) Miers | Treatment of asthma, Tuberculosis, dysentery, hyperglycaemia, cancer, fever, intestinal complaints, sleep disturbances and inflammation | Asian countries | Menispermaceae | Young Leaves | methanol:water:acetic acid (50:50:0.1, v/v/v) | (Semwal and Semwal, 2015; Gorpenchenko et al., 2017) |
| *Tinospora cordifolia* (Willd.) Hook.f. & Thomson | immunomodulation, anticancer, hepatoprotective and hypoglycemic | Indian subcontinent and China | Menispermaceae |  | ethanol | (Bajpai et al., 2016; Singh and Chaudhuri, 2017; Chowdhury, 2021) |
| *Corydalis yanhusuo* W. T. Wang | Drug addiction and pain relief, invigorate blood, invigorate qi and relieve pain | China | Papaveraceae | Tuber | 95% ethanol/methanol | (Xiao et al., 2011; Xu et al., 2015; Wu et al., 2018; Zhang et al., 2020) |
| *Corydalis ternata* (Nakai) Nakai | Anticholinesterase, antiamnesic, and anti-inflammatory activities, and analgesic effects | Asian countries | Papaveraceae | N/A | N/A | (Yun, 2014; Kim et al., 2017a) |
| *Corydalis adunca* Maxim. | Clearing away heat and toxic matter, stop bleeding, anti-inflammatory, treat gallbladder disease, remove the stone, relive pain | China | Papaveraceae | Earthnut | Methanol | (Long X.Y., 2007) |
| *Corydalis decumbens* (Thunb.) Pers. | Removing blood stasis and freeing vessel, promote qi circulation to relieve pain, treatment of paralytic stroke, headache, rheumatic arthritis and sciatica | China | Papaveraceae | Earthnut/ Rhizome/ Bulb/ Seedling | Carbon dioxide supercritical CO2 fluid extraction/95% EtOH/90% ethanol | (Wang and Peng, 2002; Shen et al., 2011; Wu et al., 2013a; Wu et al., 2013b; Huang et al., 2018) |
| *Corydalis racemosa* (Thunb.) Pers. | Antihypertensive | China | Papaveraceae | Whole plant /Root | 95% ethanol | (Wu et al., 2011) |
| *Corydalis bungeana* Turcz. | Anti-inflammatory, antibacterial activity and inhibition of the immune function of the host | China | Papaveraceae | Whole plant | 80% ethanol | (Zhai et al., 2016; Li et al., 2019) |
| *Corydalis bulbosa* (L.) DC. | N/A | Bulgaria | Papaveraceae | Tuber | MeOH | (Kiryakov and Iskrenova, 1984; Miyazawa et al., 1998) |
| *Corydalis saxicola* Bunting | Anti-inflammation, blood circulations improvement, hemostasis, and analgesia | China | Papaveraceae | Herb | Methanol | (Li et al., 2007; Kuai et al., 2020) |
| *Corydalis koidzumiana* Ohwi | N/A | N/A | Papaveraceae | N/A | N/A | N/A |
| *Corydalis cava* (L.) Schweigg. & Körte | Analgetic, sedating, narcotic, anti-inflammatory, anti-allergic and anti-tumour activities | Central and South Europe | Papaveraceae | Tuber | N/A | (Nawrot et al., 2010) |
| *Corydalis turtschaninovii* Besser | Treatment of abdominalgia, menorrhalgia, menostasia, and traumatic pain | China | Papaveraceae | N/A | Methanol and  purified water | (Tao et al., 2019; Tao et al., 2020) |
| *Corydalis ambigua* Cham. & Schltdl. | Analgesic and sedative agents | China | Papaveraceae | N/A | N/A | (Huang et al., 2021a) |
| *Glaucium corniculatum* (L.) Curtis | Memory-enhancing and neuroprotective properties | Turkey | Papaveraceae | Above-ground plant parts | Chloroform, methanol and water | (Nigdelioglu Dolanbay et al., 2021) |
| *Uvaria kweichowensis* P. T. Li | Cure inflammation and tumour | Southwest area of China | Annonaceae | Above-ground plant parts | 90% ethanol | (Zhao et al., 2006; Xu et al., 2007) |
| *Uvaria microcarpa* Champ. ex Benth. | N/A | N/A | Annonaceae | Leaves | 70% ethanol | (Liu et al., 2011) |
| *Annickia kummerae* (Engl. & Diels) Setten & Maas | Anti-plasmodia | Tanzania | Annonaceae | Leaves, Root-bark and Stem-bark | petroleum ether (PE), dichloromethane  (DCM) and methanol (MeOH) | (Malebo et al., 2013) |
| *Embelia ribes* Burm.f. | Treatment of tumors, ascites, bronchitis, jaundice, diseases of the heart and brain | India | Myrsinaceae | Fruits | hexane:dichloromethane (1:1) | (Shirole et al., 2015; Nuthakki et al., 2019) |
| *Phellodendron chinense* C.K.Schneid. | Remove damp heat, relieve consumptive fever, and cure dysentery and diarrhea | China | Berberidaceae | Cortex | N/A | (Kim et al., 2017b; Wang et al., 2019) |
| *Phellodendron amurense* Rupr. | Remove damp heat, relieve consumptive fever, and cure dysentery and diarrhea | China | Berberidaceae | Cortex | 30% ethanol | (Chen et al., 2012a) |
| *Berberis napaulensis* var. napaulensis | N/A | India（Todas of Nilgiris) | Berberidaceae | Root | ethanol (100%) | (Singh et al., 2017) |
| *Coptis deltoidea* C.Y.Cheng & P.K.Hsiao*/Coptis chinensis* Franch. | clear the damp-heat, quench the fire, and counteract the poison | Chongqing, Hubei, Guizhou, Shanxi provinces of China | Ranunculaceae | Rhizome | Liquid-liquid extraction (LLE) and protein precipitation with methanol,  acetonitrile | (Ho et al., 2014; Liu et al., 2016) |

**Table S2** Potential pharmacological action and mechanism of tetrahydropalmatine.

| **Pharmacological effect** | **Cell lines/model** | **Activity/mechanism(s)** | **Application** | **Dosage** | **References** |
| --- | --- | --- | --- | --- | --- |
| **Analgesic activity** | Partial sciatic nerve ligation (PSNL)-reduced chronic neuropathic pain mouse model | Antagonizes D2R | In vivo | 5 and 10 mg/kg | (Huang et al., 2021b) |
|  | Chronic constriction injury mice | Modulates spinal Sig-1R | In vivo | 0.2, 2, 20, 200nmol | (Kang et al., 2016) |
|  | Intraplantar injection of complete Freund's adjuvant | Increases dopamine D1 receptor-mediated dopaminergic transmission | In vivo | 1~4 mg/kg, i.p. | (Zhou et al., 2016) |
|  | Rats with bone cancer pain caused by tumor cell implantation (TCI) | Inhibits the activation of microglial cells and the increase of TNF-alpha and IL-18 | In vivo | 20, 40 and 60 mg/kg | (Zhang et al., 2015) |
|  | Oxaliplatin-induced mouse model in neuropathic pain | Modulates dopamine D-1 receptor | In vivo | 1~4 mg/kg, i.p. | (Guo et al., 2014) |
|  | Pretreatment of dl-THP in rats | Modulates the suprasipinal level | In vivo | 20, 40, 60 mg/kg, body weight | (Cao et al., 2011) |
|  | Adult female rats | Decreases immunoreactivity to all mediators involved in central sensitization and to HDAC2 in DRG, to TrkA and CGRP in ectopic endometrium, and to CGRP in eutopic endometrium | In vivo | 70 and 140 mg/kg | (Zhao et al., 2011) |
|  | Tamoxifen-induced female ICR mice Adenomyosis | Inhibits myometrial infiltration; Improves generalized hyperalgesia; Reduces the amplitude and irregularity of uterine contractions. | In vivo | 10 and 20 mg/kg | (Mao et al., 2011) |
|  | Wistar rat uterine contraction model | Inhibits the contraction of isolated uteri caused by Ach, PGF (2 alpha), and oxytocin; Affected the levels of NO, activation of NF-kappa B, up-regulated the expression of i-kappa B and down-regulated the expression of both iNOS and COX-2 | In vivo | 0.07 g/kg | (Chen et al., 2013) |
|  | Female Sprague-Dawley rats aged 6-7 weeks | Decreases MMP-2 and MMP-9, increasing TIMP-1; Promotes E-cadherin, and attenuated N-cadherin, Vimentin, Snail, Slug, ZEB1, ZEB2, Twist. | In vivo | N/A | (Chen et al., 2018) |
| **Antiaddiction activity** | METH-induced mice CPP | Inhibits the rewarding properties | In vivo | 1.25, 2.5, 5.0, 10.0 and 20.0 mg/kg | (Su et al., 2013) |
|  | Naive rat brain | Selectively activates the key brain regions of the dopaminergic, serotonergic and noradrenergic systems | In vivo | 5, 10, 20 and 40 mg/kg | (Liu et al., 2012) |
|  | Morphine-induced rats CPP;Aale adult Sprague-Dawley rats | Inhibits D-2R down-regulation and GluA1 AMPA receptor up-regulation | In vivo | 1.25, 2.5, 5 mg/kg | (Jiang et al., 2020) |
|  | Rat model of morphine-dependence | Antagonism of the dopamine autoreceptor | In vivo | 5mg/kg | (Ahn et al., 2020) |
|  | Human embryonic kidney-293 cells (HEK293) | Antagonistic potency on dopamine D1 receptors | In vitro | 0.6437uM | (Wu et al., 2018) |
|  | Rats | Modulates 5-HT neuronal activity and dopamine D3 receptor expression | In vivo | 10 and 15 mg/kg | (Yun, 2014) |
|  | Wistar rats | Up-regulates the level of plasma beta-endorphin and hypothalamic POMC | In vivo | 3, 5 and 10mg/kg | (Sushchyk et al., 2016) |
|  | Human neuronal nAChRs/Rats trained to self-administer nicotine | Blocks neuronal alpha 4 beta 2-nAChR function; Increases extracellular dopamine (DA) levels in the nucleus accumbens shell (nAcb) | In vitro and In vivo | 1.8×10^−5^M; 3 and 5 mg/kg | (Huang et al., 2021a) |
|  | L-THP treated mice | Modulates D2R-mediated PICA signaling in the CPu | In vivo | 2.5, 5, and 10 mg/kg | (Kim et al., 2013) |
|  | METH-induced mice CPP | Inhibits ERK phosphorylation in NAc and PFc; Decreases level of NAc and PFc | In vivo | 10.0mg/kg | (Su et al., 2020) |
|  | Ketamine-induced rats CPP | Inhibits ERK and CREB phosphorylation in Hip and CPu | In vivo | 10 and 20mg/kg | (Du et al., 2017) |
|  | Fentanyl-induced rewarding behavior through conditioned place preference (CPP) in mice | Suppresses ERK and CREB phosphorylation in the Hip, NAc, and PFC of mice | In vivo | 5.0 and 10.0 mg/kg | (Du et al., 2021) |
|  | METH-induced mice locomotor sensitization | Inhibits ERK1/2 phosphorylation in the NAc and CPu | In vivo | 5 and 10 mg/kg | (Zhao et al., 2014) |
|  | Ketamine-induced learning and memory impairment in mice | Inhibits oxidative stress, inflammation and acetylcholinesterase activity  decreases acetylcholine levels | In vivo | 20, 40 and 80 mg/kg | (Zhang et al., 2018) |
|  | Mice | Reverses the impairment of acuquisition and retention of spatial memory | In vivo | 10.0 and 20 mg/kg | (Cao et al., 2018) |
|  | Male C57BL/6 mice | Modulates ERK1/2 expression in the PFC | In vivo | 5 and 10 mg/kg | (Chen et al., 2012b) |
|  | Male Sprague-Dawley rats | Decreases METH self-administration; Inhibits METH-induced reinstatement of METH-seeking behaviors; Conserves locomotor activity | In vivo | 0.00, 1.25, 2.50 and 5.00 mg/kg, i.p. | (Gong et al., 2016) |
|  | Abstinent rats | Inhibits heroin-induced reinstatement of heroin-seeking behavior;  Conserves locomotion | In vivo | 0, 1.25, 2.5 and 5 mg/kg, i.p. | (Yue et al., 2012) |
|  | Male Sprague-Dawley rats | Conserves non-specific motor | In vivo | 3.0 or 10.0 mg/kg | (Figueroa-Guzman et al., 2011) |
| **Anti-inflammatory activity** | Bee Venom (BV)-induced persistent spontaneous pain-related behaviors in rats | Down-regulates P2X3 receptors and TRPV1 | In vivo | 20, 40, 60 mg/kg | (Wang et al., 2021) |
|  | Limb ischemia/reperfusion rat model;  Male Sprague–Dawley rats | Inhibits PI3K/AKT/mTOR activity; | In vivo | 10, 20 and 40 mg/kg | (Wen et al., 2020) |
|  | BALB/c mice | Inhibits the activation of ERK/NF-kappa B signaling pathway | In vivo | 20 and 40 mg/kg | (Yu et al., 2019) |
|  | In vivo: lipopolysaccharide-induced DIC model  In vitro: RAW 264.7 macrophages with LPS model | Inhibits TNF-alpha expression; Supresses the activation of NF-kappa B signaling pathway; Modifies coagulation indexes; Reduces the inflammatory cytokine production | In vivo and in vitro | vivo:30 and 60 mg/kg  vitro:60 or 120 mM | (Zhi et al., 2020) |
|  | Rat model of myocardial ischaemia-reperfusion  injury | Decreases the accumulation of inflammatory factors, including TNF-alpha and MPO; Inhibits the extent of apoptosis | In vivo | 10, 20 or 40 mg/kg b.w. | (Han et al., 2012) |
|  | A (ConA-) induced hepatitis in Balb/c mice | Inhibits apoptosis and autophagy via the TRAF6/JNK pathway | In vivo | 20 or 40mg/kg | (Yu et al., 2018) |
|  | High-fat diet (HFD)-fed golden hamsters | Inhibits the accumulation of hepatic lipid | In vivo | 6.3, 12.6 and 25.2 g/kg/day | (Sun et al., 2018a) |
|  | Human umbilical vein endothelial cells (HUVECs) | Inhibits monocyte adhesion to vascular endothelial cell; Downregulates ICAM-1 and VCAM-1 in vascular endothelial cell | In vitro | 3, 10, 30 μmol/L | (Yang et al., 2015) |
|  | 4-5 weeks old BALB/c mice of either sex; Japanese encephalitis virus strain GP-78 infected mouse model | Decreases the level of viral population, caspase-2 expression, reactive oxygen and nitrogen species, microglial cells and proinflammatory mediators, stress linked protein molecules and neuronal apoptosis | In vivo | 2mg/kg | (Lixia et al., 2018) |
|  | Sprague-Dawley rats; Irradiation induced lung injuries in rats | Inhibits the pulmonary cells apoptosis; Decreases BALF cells recruitment, BALF protein levels and collagen content of lung tissues | In vivo | 40mg/kg | (Yu et al., 2016) |
|  | Sprague-Dawley rats | Inhibits inflammation, oxidative stress; Conserves vascular smooth muscle cells (VSMCs) | In vivo | 15mg/kg | (Wang et al., 2018) |
|  | D-gal induced memory impairment in rats | Decreases MDA, NO; Increases GSH, SOD, CAT, GPx; Reverses the abnormality of ACh and AChE; Inhibits the expression of NF-KB and GFAP | In vivo | 20, 40, 80mg/kg/d | (Qu et al., 2016) |
| **Neuroprotective activity** | Ischemic stroke model of Sprague Dawley male rats | Inhibits c-Abl overexpression | In vivo | 12.5, 25, and 50 mg/kg | (Sun et al., 2018b) |
|  | HEK293 cells | Inhibits the delayed rectifier Kv1.5 channels | In vitro | 10 and 50uM | (Li et al., 2017) |
|  | Rat dorsal root ganglion (DRG) neurons | Inhibits the functional activity of ASICs in dissociated primary sensory neurons; Relieves acidosis-evoked pain in vivo | In vitro  In vivo | 10^-5^M | (Liu et al., 2015) |
|  | Single prolonged stress (SPS)-reduced rats of anxiety and depression | Reverses impairments of traumatic stress; Inhibits the decrease in neuropeptide Y (NPY) and the increase in corticotrophin-releasing factor (CRF) expression in the hypothalamus | In vivo | 10, 20, 50 mg/kg body weight | (Lee et al., 2014a) |
|  | Male Sprague-Dawley rats; Post-traumatic stress disorder-induced changes in rat | Changes transcriptional fold of dopamine, serotonin, acetylcholine, and gamma-aminobutyric acid neurotransmitter systems | In vivo | 20mg/kg | (Ceremuga et al., 2013) |
|  | Cisplatin-resistant A2780/DDP cell line | Modulates miR-93/PTEN/AKT pathway | In vitro | 0,50,100,150 and 200 µM | (Gong et al., 2019) |
|  | In vivo: tumor-bearing nude mice  In vitro: Mouse primary renal tubular cells (mPRTCs) and human primary renal tubular cells (hPRTCs) | Selectively inhibits OCT2; Maintains Pt concentration and Pt’s antitumor efficacy; Decreases cisplatin accumulation and cisplatin-induced cytotoxicity in human primary renal tubular cells | In vivo and in vitro | 5~40mg/kg | (Li et al., 2020) |
|  | ER alpha (+) BCa cells | Induces cell cycle arrest; Increases sensitivity to tamoxifen and fulvestrant;  Promotes ER alpha degradation | In vitro | 25, 50, 100, 200 uM | (Xia et al., 2020) |
| **Other pharmacological activities** | MDCK-hOCT2/hMATE1 and MDCK-hOCT2/pcDNA3.1 cells | Reduces NC accumulation and cytotoxicity in MDCK-hOCT2, MDCK-hOCT2/hMATE1 and rPCPT cells | In vitro | 50 μM | (Li et al., 2016) |
|  | MDCK-hOCT1 or MDCK-hOCT3 and mock cells | Reduces the uptake of NC in MDCK-hOCT1 cells, MDCK-hOCT3 cells, and rat primary hepatocytes | In vitro | 50 mM | (Li et al., 2014) |
|  | Aorta of Male Wistar rats | Modulates PI3K/Akt/eNOS/NO/cGMP signaling pathway, Ca2+ channels and K+ channels | In vitro | 1, 3, 10, and 30, 100 µM | (Zhou et al., 2019) |
|  | In vitro: rat’s aorta  In vivo: Male Wistar rats, Wistar Kyoto rats (WKY) and spontaneous hypertensive rats (SHR) | Modulates the activation of NO/cGMP pathway and calcium channel blockade | In vivo and in vitro | vivo: 20, 40 and 80 mg/kg  vitro: 18.8, 37.6, 75.2, 150.4, 300.8 and 601.6 µg/mL | (Qu et al., 2015) |
|  | Quail chick chorioallantoic membrane (qCAM); HUVECs | Effects citrulline to arginine flux, arginine biosynthesis, and endothelial VEGFR2 expression sequentially | In vitro | 16, 32, and 64 µg  20, 40, 80 µM | (Cui et al., 2021) |
|  | Myoblast C2C12 cells and embryonic fifibroblast 10T1/2 cells | Up-regulates p38MAPK and Akt; Modifies MyoD activation | In vitro | 100–1000 nM | (Lee et al., 2014b) |
|  | Mouse 3T3-L1 preadipocytes | Modulates the AMPK pathway | In vitro | 0, 10, 20 or 40 µM | (Piao et al., 2017) |
|  | In vitro: activated LX2 cell model induced by TGF-beta 1  In Vivo: mouse hepatic fibrosis models (male C57 mice) | Inhibits ECM deposition and HSCs autophagy; Modulates PPAR gamma/NF-kappa B and TGF-beta 1/Smad pathway | In vitro and in vivo | 20 and 40 mg/kg | (Yu et al., 2021) |
|  | P.falciparum-type W2 | Antiplasmodial activity | In vitro | N/A | (Bory et al., 2013) |
|  | Trypanosoma brucei rhodesiense STIB 900 strain | Anti-trypanosomal activity | In vitro | N/A | (Malebo et al., 2013) |
|  | Strain of Plasmodium falciparum type W2 | Antiplasmodial activity | In vitro | N/A | (Baghdikian et al., 2013) |
|  | Cestode parasite, Raillietina echinobothrida | Interacts with active site residues of PEPCK from the parasite | In vitro | 0·25 mM | (Dutta et al., 2016) |
|  | Eight plant pathogenic fungi species, Rhizoctonia solani, Botrytis cinerea, Fusarium graminearum, Mycosphaerlla melonis, Fusarium oxysporum f. sp. Vasinfectum, Phyllosticta zeae, Sclerotinia sclerotiorum and Magnaporthe oryzae | Anti-phytopathogenic activity | In vitro | N/A | (Zhao et al., 2019) |

**Table S3** Tissue distribution of potential targets of tetrahydropalmatine.

| **Term** | **Count** | **%** | **P-Value** |
| --- | --- | --- | --- |
| Brain | 60 | 56.6 | 3.60E-03 |
| Blood | 11 | 10.4 | 8.60E-03 |
| Fetal brain | 11 | 10.4 | 1.10E-02 |
| Hippocampus | 9 | 8.5 | 4.00E-03 |
| Platelet | 9 | 8.5 | 1.40E-02 |
| Peripheral blood | 4 | 3.8 | 3.30E-02 |
| Corpus striatum | 3 | 2.8 | 2.90E-04 |
| Fetal lung | 3 | 2.8 | 5.30E-02 |
| Myeloid | 2 | 1.9 | 5.80E-02 |

**Table S4** Disease enrichment classification of potential targets of tetrahydropalmatine (Count%≥30).

| **Term** | **Count** | **%** | **P-Value** |
| --- | --- | --- | --- |
| Metabolic | 78 | 73.6 | 1.60E-12 |
| Pharmacogenomic | 65 | 61.3 | 9.40E-20 |
| Psych | 64 | 60.4 | 1.30E-26 |
| Cancer | 63 | 59.4 | 1.60E-14 |
| Chem-dependency | 60 | 56.6 | 4.30E-10 |
| Neurological | 55 | 51.9 | 2.00E-11 |
| Cardiovascular | 54 | 50.9 | 7.10E-05 |
| Immune | 49 | 46.2 | 1.10E-07 |
| Renal | 33 | 31.1 | 2.30E-08 |
| Reproduction | 33 | 31.1 | 1.10E-12 |

**References**

Ahn, S., Nesbit, M.O., Zou, H., Vacca, G., Axerio-Cilies, P., Van Sung, T., et al. (2020). Neural bases for attenuation of morphine withdrawal by Heantos-4: role of l-tetrahydropalmatine. *Sci Rep* 10(1)**,** 21275. doi: 10.1038/s41598-020-78083-x.

Baghdikian, B., Mahiou-Leddet, V., Bory, S., Bun, S.S., Dumetre, A., Mabrouki, F., et al. (2013). New antiplasmodial alkaloids from Stephania rotunda. *J Ethnopharmacol* 145(1)**,** 381-385. doi: 10.1016/j.jep.2012.10.052.

Bajpai, V., Singh, A., Chandra, P., Negi, M.P., Kumar, N., and Kumar, B. (2016). Analysis of phytochemical variations in dioecious Tinospora cordifolia stems using HPLC/QTOF MS/MS and UPLC/QqQLIT -MS/MS. *Phytochem Anal* 27(2)**,** 92-99. doi: 10.1002/pca.2601.

Bartley, J.P., Baker, L.T., and Carvalho, C.F. (1994). Alkaloids of Stephania bancroftii. *Phytochemistry* 36(5)**,** 1327-1331.

Bory, S., Bun, S.S., Baghdikian, B., Dumètre, A., Hutter, S., Mabrouki, F., et al. (2013). HPLC analysis of Stephania rotunda extracts and correlation with antiplasmodial activity. *Phytother Res* 27(2)**,** 278-284. doi: 10.1002/ptr.4710.

Cao, F.L., Shang, G.W., Wang, Y., Yang, F., Li, C.L., and Chen, J. (2011). Antinociceptive effects of intragastric DL-tetrahydropalmatine on visceral and somatic persistent nociception and pain hypersensitivity in rats. *Pharmacol Biochem Behav* 100(1)**,** 199-204. doi: 10.1016/j.pbb.2011.08.016.

Cao, G., Zhang, Y., Zhu, L., Zhu, J., Zhao, N., Dong, N., et al. (2018). The inhibitory effect of levo-tetrahydropalmatine on the methamphetamine-induced spatial memory impairment in mice. *Neurosci Lett* 672**,** 34-39. doi: 10.1016/j.neulet.2018.02.018.

Ceremuga, T.E., Shellabarger, P., Persson, T., Fanning, M., Galey, P., Robinson, D., et al. (2013). Effects of tetrahydropalmatine on post-traumatic stress disorder-induced changes in rat brain gene expression. *J Integr Neurosci* 12(4)**,** 513-528. doi: 10.1142/s0219635213500313.

Chen, X., Cao, Y., Lv, D., Zhu, Z., Zhang, J., and Chai, Y. (2012a). Comprehensive two-dimensional HepG2/cell membrane chromatography/monolithic column/time-of-flight mass spectrometry system for screening anti-tumor components from herbal medicines. *J Chromatogr A* 1242**,** 67-74. doi: 10.1016/j.chroma.2012.04.034.

Chen, Y., Cao, Y., Xie, Y., Zhang, X., Yang, Q., Li, X., et al. (2013). Traditional Chinese medicine for the treatment of primary dysmenorrhea: how do Yuanhu painkillers effectively treat dysmenorrhea? *Phytomedicine* 20(12)**,** 1095-1104. doi: 10.1016/j.phymed.2013.05.003.

Chen, Y., Wei, J., Zhang, Y., Sun, W., Li, Z., Wang, Q., et al. (2018). Anti-endometriosis Mechanism of Jiawei Foshou San Based on Network Pharmacology. *Front Pharmacol* 9**,** 811. doi: 10.3389/fphar.2018.00811.

Chen, Y.J., Liu, Y.L., Zhong, Q., Yu, Y.F., Su, H.L., Toque, H.A., et al. (2012b). Tetrahydropalmatine protects against methamphetamine-induced spatial learning and memory impairment in mice. *Neurosci Bull* 28(3)**,** 222-232. doi: 10.1007/s12264-012-1236-4.

Chowdhury, P. (2021). In silico investigation of phytoconstituents from Indian medicinal herb 'Tinospora cordifolia (giloy)' against SARS-CoV-2 (COVID-19) by molecular dynamics approach. *J Biomol Struct Dyn* 39(17)**,** 6792-6809. doi: 10.1080/07391102.2020.1803968.

Cui, H., Yang, X., Wang, Z., Li, G., Li, L., Huo, S., et al. (2021). Tetrahydropalmatine triggers angiogenesis via regulation of arginine biosynthesis. *Pharmacol Res* 163**,** 105242. doi: 10.1016/j.phrs.2020.105242.

Dary, C., Baghdikian, B., Kim, S., Mabrouki, F., Hul, S., Jabbour, F., et al. (2017a). Optimization of ultrasound-assisted extraction of bioactive alkaloids from Stephania cambodica using response surface methodology. *Comptes Rendus Chimie* 20(11-12)**,** 996-1005. doi: 10.1016/j.crci.2017.09.004.

Dary, C., Bun, S.S., Herbette, G., Mabrouki, F., Bun, H., Kim, S., et al. (2017b). Chemical profiling of the tuber of Stephania cambodica Gagnep. (Menispermaceae) and analytical control by UHPLC-DAD. *Nat Prod Res* 31(7)**,** 802-809. doi: 10.1080/14786419.2016.1247077.

Desgrouas, C., Taudon, N., Bun, S.S., Baghdikian, B., Bory, S., Parzy, D., et al. (2014). Ethnobotany, phytochemistry and pharmacology of Stephania rotunda Lour. *J Ethnopharmacol* 154(3)**,** 537-563. doi: 10.1016/j.jep.2014.04.024.

Du, K., Wang, Z., Zhang, H., Zhang, Y., Su, H., Wei, Z., et al. (2021). Levo-tetrahydropalmatine attenuates the acquisition of fentanyl-induced conditioned place preference and the changes in ERK and CREB phosphorylation expression in mice. *Neurosci Lett* 756**,** 135984. doi: 10.1016/j.neulet.2021.135984.

Du, Y., Du, L., Cao, J., Hölscher, C., Feng, Y., Su, H., et al. (2017). Levo-tetrahydropalmatine inhibits the acquisition of ketamine-induced conditioned place preference by regulating the expression of ERK and CREB phosphorylation in rats. *Behav Brain Res* 317**,** 367-373. doi: 10.1016/j.bbr.2016.10.001.

Dutta, A.K., Ramnath, Tandon, V., and Das, B. (2016). Biocomputational analysis of phosphoenolpyruvate carboxykinase from Raillietina echinobothrida, a cestode parasite, and its interaction with possible modulators. *Parasitology* 143(3)**,** 300-313. doi: 10.1017/s0031182015001742.

Figueroa-Guzman, Y., Mueller, C., Vranjkovic, O., Wisniewski, S., Yang, Z., Li, S.J., et al. (2011). Oral administration of levo-tetrahydropalmatine attenuates reinstatement of extinguished cocaine seeking by cocaine, stress or drug-associated cues in rats. *Drug Alcohol Depend* 116(1-3)**,** 72-79. doi: 10.1016/j.drugalcdep.2010.11.023.

Gong, J., Xing, C., Wang, L.Y., Xie, S.S., and Xiong, W.D. (2019). L-Tetrahydropalmatine enhances the sensitivity of human ovarian cancer cells to cisplatin via microRNA-93/PTEN/Akt cascade. *J buon* 24(2)**,** 701-708.

Gong, X., Yue, K., Ma, B., Xing, J., Gan, Y., Wang, D., et al. (2016). Levo-tetrahydropalmatine, a natural, mixed dopamine receptor antagonist, inhibits methamphetamine self-administration and methamphetamine-induced reinstatement. *Pharmacol Biochem Behav* 144**,** 67-72. doi: 10.1016/j.pbb.2016.01.010.

Gorpenchenko, T., Y., Grigorchuk, V., P., et al. (2017). Stepharine production in morphogenic cell cultures of Stephania glabra (ROXB.) Miers. *PLANT CELL TISSUE AND ORGAN CULTURE*.

Guo, Z., Man, Y., Wang, X., Jin, H., Sun, X., Su, X., et al. (2014). Levo-tetrahydropalmatine attenuates oxaliplatin-induced mechanical hyperalgesia in mice. *Sci Rep* 4**,** 3905. doi: 10.1038/srep03905.

Han, Y., Zhang, W., Tang, Y., Bai, W., Yang, F., Xie, L., et al. (2012). l-Tetrahydropalmatine, an active component of Corydalis yanhusuo W.T. Wang, protects against myocardial ischaemia-reperfusion injury in rats. *PLoS One* 7(6)**,** e38627. doi: 10.1371/journal.pone.0038627.

Ho, C.E., Goh, Y.L., and Zhang, C. (2014). From prejudice to evidence: the case of rhizoma coptidis in singapore. *Evid Based Complement Alternat Med* 2014**,** 871720. doi: 10.1155/2014/871720.

Huang, Q., Chen, J., Zhang, W., Zhou, B., Zhang, C., Gerwick, W.H., et al. (2018). Alkaloids from Corydalis decumbens suppress neuronal excitability in primary cultures of mouse neocortical neurons. *Phytochemistry* 150**,** 85-92. doi: 10.1016/j.phytochem.2018.03.006.

Huang, Y.B., Ma, Z.G., Zheng, C., Ma, X.K., Taylor, D.H., Gao, M., et al. (2021a). Levo-tetrahydropalmatine inhibits α4β2 nicotinic receptor response to nicotine in cultured SH-EP1 cells. *Acta Pharmacol Sin*. doi: 10.1038/s41401-021-00709-1.

Huang, Y.G., Flaherty, S.J., Pothecary, C.A., Foster, R.G., Peirson, S.N., and Vyazovskiy, V.V. (2021b). The relationship between fasting-induced torpor, sleep, and wakefulness in laboratory mice. *Sleep* 44(9). doi: 10.1093/sleep/zsab093.

Jiang, W.N., Jing, X., Li, M., Deng, H., Jiang, T., Xiong, K.Z., et al. (2020). Corydaline and l-tetrahydropalmatine attenuate morphine-induced conditioned place preference and the changes in dopamine D(2) and GluA1 AMPA receptor expression in rats. *Eur J Pharmacol* 884**,** 173397. doi: 10.1016/j.ejphar.2020.173397.

Kang, D.W., Moon, J.Y., Choi, J.G., Kang, S.Y., Ryu, Y., Park, J.B., et al. (2016). Antinociceptive Profile of Levo-tetrahydropalmatine in Acute and Chronic Pain Mice Models: Role of spinal sigma-1 receptor. *Sci Rep* 6**,** 37850. doi: 10.1038/srep37850.

Kim, T., Hinton, D.J., Johng, S., Wang, J.B., and Choi, D.S. (2013). Levo-tetrahydropalmatine decreases ethanol drinking and antagonizes dopamine D2 receptor-mediated signaling in the mouse dorsal striatum. *Behav Brain Res* 244**,** 58-65. doi: 10.1016/j.bbr.2013.01.028.

Kim, Y.J., Lim, H.S., Kim, Y., Lee, J., Kim, B.Y., and Jeong, S.J. (2017a). Neuroprotective Effect of Corydalis ternata Extract and Its Phytochemical Quantitative Analysis. *Chem Pharm Bull (Tokyo)* 65(9)**,** 826-832. doi: 10.1248/cpb.c17-00300.

Kim, Y.J., Lim, H.S., Kim, Y., Lee, J., Kim, B.Y., and Jeong, S.J. (2017b). Phytochemical Quantification and the In Vitro Acetylcholinesterase Inhibitory Activity of Phellodendron chinense and Its Components. *Molecules* 22(6). doi: 10.3390/molecules22060925.

Kiryakov, H.G., and Iskrenova, E.S. (1984). Minor Alkaloids of Corydalis bulbosa, Structure of Bulbodione. *Planta Med* 50(2)**,** 136-138. doi: 10.1055/s-2007-969652.

Kongkiatpaiboon, S., Duangdee, N., Prateeptongkum, S., and Chaijaroenkul, W. (2016). Acetylcholinesterase Inhibitory Activity of Alkaloids Isolated from Stephania venosa. *Nat Prod Commun* 11(12)**,** 1805-1806.

Kuai, C.P., Ju, L.J., Hu, P.P., and Huang, F. (2020). Corydalis saxicola Alkaloids Attenuate Cisplatin-Induced Neuropathic Pain by Reducing Loss of IENF and Blocking TRPV1 Activation. *Am J Chin Med* 48(2)**,** 407-428. doi: 10.1142/s0192415x20500214.

Le, P.M., Srivastava, V., Nguyen, T.T., Pradines, B., Madamet, M., Mosnier, J., et al. (2017). Stephanine from Stephania venosa (Blume) Spreng Showed Effective Antiplasmodial and Anticancer Activities, the Latter by Inducing Apoptosis through the Reverse of Mitotic Exit. *Phytother Res* 31(9)**,** 1357-1368. doi: 10.1002/ptr.5861.

Lee, B., Sur, B., Yeom, M., Shim, I., Lee, H., and Hahm, D.H. (2014a). L-tetrahydropalmatine ameliorates development of anxiety and depression-related symptoms induced by single prolonged stress in rats. *Biomol Ther (Seoul)* 22(3)**,** 213-222. doi: 10.4062/biomolther.2014.032.

Lee, S.J., Yoo, M., Go, G.Y., Hwang, J., Lee, H.G., Kim, Y.K., et al. (2014b). Tetrahydropalmatine promotes myoblast differentiation through activation of p38MAPK and MyoD. *Biochem Biophys Res Commun* 455(3-4)**,** 147-152. doi: 10.1016/j.bbrc.2014.10.115.

Li, C., Li, L., Yi, Y., Wang, W., Yuan, J., Tan, F., et al. (2020). L-tetrahydropalmatine attenuates cisplatin-induced nephrotoxicity via selective inhibition of organic cation transporter 2 without impairing its antitumor efficacy. *Biochem Pharmacol* 177**,** 114021. doi: 10.1016/j.bcp.2020.114021.

Li, H.L., Zhang, W.D., Han, T., Zhang, C., Liu, R.H., and Chen, H.S. (2007). Tetrahydroprotoberberine alkaloids from Corydalis saxicola. *Chemistry of Natural Compounds* 43(2)**,** 173-175.

Li, K., Pi, M.S., and Li, X.T. (2017). The inhibitory effects of levo-tetrahydropalmatine on rat Kv1.5 channels expressed in HEK293 cells. *Eur J Pharmacol* 809**,** 105-110. doi: 10.1016/j.ejphar.2017.05.020.

Li, L., Tu, M., Yang, X., Sun, S., Wu, X., Zhou, H., et al. (2014). The contribution of human OCT1, OCT3, and CYP3A4 to nitidine chloride-induced hepatocellular toxicity. *Drug Metab Dispos* 42(7)**,** 1227-1234. doi: 10.1124/dmd.113.056689.

Li, L.P., Song, F.F., Weng, Y.Y., Yang, X., Wang, K., Lei, H.M., et al. (2016). Role of OCT2 and MATE1 in renal disposition and toxicity of nitidine chloride. *Br J Pharmacol* 173(16)**,** 2543-2554. doi: 10.1111/bph.13537.

Li,M.Z., Zhang,H., and Zhu, F.X. (2019). Study on the chemical constituents of Corydalis bungeana, West China Journal of Pharmaceutical Sciences 34(3), 7.

Liu, L., Wang, Z.B., Song, Y., Yang, J., Wu, L.J., Yang, B.Y., et al. (2016). Simultaneous Determination of Eight Alkaloids in Rat Plasma by UHPLC-MS/MS after Oral Administration of Coptis deltoidea C. Y. Cheng et Hsiao and Coptis chinensis Franch. *Molecules* 21(7). doi: 10.3390/molecules21070913.

Liu, T.T., Qu, Z.W., Qiu, C.Y., Qiu, F., Ren, C., Gan, X., et al. (2015). Inhibition of acid-sensing ion channels by levo-tetrahydropalmatine in rat dorsal root ganglion neurons. *J Neurosci Res* 93(2)**,** 333-339. doi: 10.1002/jnr.23484.

Liu, X., Yang, Z., Li, R., Xie, J., Yin, Q., Bloom, A.S., et al. (2012). Responses of dopaminergic, serotonergic and noradrenergic networks to acute levo-tetrahydropalmatine administration in naïve rats detected at 9.4 T. *Magn Reson Imaging* 30(2)**,** 261-270. doi: 10.1016/j.mri.2011.09.006.

Liu, X.R., Chen, Z., Li, X.R., Xu, Q.M., and Yang, S.L. (2011). Chemical constituents from leaves of Uvaria microcarpa, Chinese Traditional and Herbal Drugs 42(11), 3.

Lixia, H., Jun, C., Song, H., FaHu, Y., and Jinwen, T. (2018). Neuroprotective effect of (-)-tetrahydropalmatine in Japanese encephalitis virus strain GP-78 infected mouse model. *Microb Pathog* 114**,** 197-203. doi: 10.1016/j.micpath.2017.11.047.

Long, X.Y. (2007). etermination of Tetrahydropalmatine in Corydalis adunca Maxim by HPLC Method, *Journal of Liaoning University of Traditional Chinese Medicine* 9(5), 163-164.

Ma, Y.S., Yu, H., Li, Y.Y., Yan, H., and Cheng, X. (2008). A study of genetic structure of Stephania yunnanensis (Menispermaceae) by DALP. *Biochem Genet* 46(3-4)**,** 227-240. doi: 10.1007/s10528-008-9146-x.

Malebo, H.M., Wenzler, T., Cal, M., Swaleh, S.M., Omolo, M.O., Hassanali, A., et al. (2013). Anti-protozoal activity of aporphine and protoberberine alkaloids from Annickia kummeriae (Engl. & Diels) Setten & Maas (Annonaceae). *BMC Complement Altern Med* 13**,** 48. doi: 10.1186/1472-6882-13-48.

Mao, X., Wang, Y., Carter, A.V., Zhen, X., and Guo, S.W. (2011). The retardation of myometrial infiltration, reduction of uterine contractility, and alleviation of generalized hyperalgesia in mice with induced adenomyosis by levo-tetrahydropalmatine (l-THP) and andrographolide. *Reprod Sci* 18(10)**,** 1025-1037. doi: 10.1177/1933719111404610.

Miyazawa, M., Yoshio, K., Ishikawa, Y., and Kameoka, H. (1998). Insecticidal alkaloids from Corydalis bulbosa against Drosophila melanogaster. *Journal of Agricultural and Food Chemistry* 46(5)**,** 1914-1919. doi: 10.1021/jf9701897.

Nawrot, R., Wolun-Cholewa, M., Bialas, W., Wyrzykowska, D., Balcerkiewicz, S., and Gozdzicka-Jozefiak, A. (2010). Cytotoxic activity of proteins isolated from extracts of Corydalis cava tubers in human cervical carcinoma HeLa cells. *BMC Complement Altern Med* 10**,** 78. doi: 10.1186/1472-6882-10-78.

Nigdelioglu Dolanbay, S., Kocanci, F.G., and Aslim, B. (2021). Neuroprotective effects of allocryptopine-rich alkaloid extracts against oxidative stress-induced neuronal damage. *Biomed Pharmacother* 140**,** 111690. doi: 10.1016/j.biopha.2021.111690.

Nuthakki, V.K., Sharma, A., Kumar, A., and Bharate, S.B. (2019). Identification of embelin, a 3-undecyl-1,4-benzoquinone from Embelia ribes as a multitargeted anti-Alzheimer agent. *Drug Dev Res* 80(5)**,** 655-665. doi: 10.1002/ddr.21544.

Piao, G.C., Liu, G.C., Jin, X.J., Jin, D., and Yuan, H.D. (2017). Tetrahydropalmatine inhibits lipid accumulation through AMPK signaling pathway in 3T3‑L1 adipocytes. *Mol Med Rep* 15(6)**,** 3912-3918. doi: 10.3892/mmr.2017.6473.

Qu, Z., Zhang, J., Yang, H., Huo, L., Gao, J., Chen, H., et al. (2016). Protective effect of tetrahydropalmatine against d-galactose induced memory impairment in rat. *Physiol Behav* 154**,** 114-125. doi: 10.1016/j.physbeh.2015.11.016.

Qu, Z., Zhang, J., Huo, L., Chen, H., Li, H., Fan, Y., et al. (2015). Antihypertensive and vasorelaxant effects of Rhizoma corydalis and its active component tetrahydropalmatine via NO/cGMP pathway and calcium channel blockade in isolated rat thoracic aorta. *Rsc Advances* 5(114)**,** 94130-94143.

Semwal, D.K., and Semwal, R.B. (2015). Efficacy and safety of Stephania glabra: an alkaloid-rich traditional medicinal plant. *Nat Prod Res* 29(5)**,** 396-410. doi: 10.1080/14786419.2014.955487.

Shen, Y., Han, C., Jiang, Y., Zhou, X., Zhu, Z., and Lei, X. (2011). Rapid quantification of four major bioactive alkaloids in Corydalis decumbens (Thunb.) Pers. by pressurised liquid extraction combined with liquid chromatography-triple quadrupole linear ion trap mass spectrometry. *Talanta* 84(4)**,** 1026-1031. doi: 10.1016/j.talanta.2011.03.001.

Shirole, R.L., Shirole, N.L., and Saraf, M.N. (2015). Embelia ribes ameliorates lipopolysaccharide-induced acute respiratory distress syndrome. *J Ethnopharmacol* 168**,** 356-363. doi: 10.1016/j.jep.2015.03.009.

Singh, A., Bajpai, V., Kumar, S., Singh Rawat, A.K., and Kumar, B. (2017). Analysis of isoquinoline alkaloids from Mahonia leschenaultia and Mahonia napaulensis roots using UHPLC-Orbitrap-MS(n) and UHPLC-QqQ(LIT)-MS/MS. *J Pharm Anal* 7(2)**,** 77-86. doi: 10.1016/j.jpha.2016.10.002.

Singh, D., and Chaudhuri, P.K. (2017). Chemistry and Pharmacology of Tinospora cordifolia. *Nat Prod Commun* 12(2)**,** 299-308.

Su, H., Sun, T., Wang, X., Du, Y., Zhao, N., Zhu, J., et al. (2020). Levo-tetrahydropalmatine attenuates methamphetamine reward behavior and the accompanying activation of ERK phosphorylation in mice. *Neurosci Lett* 714**,** 134416. doi: 10.1016/j.neulet.2019.134416.

Su, H.L., Zhu, J., Chen, Y.J., Zhao, N., Han, W., Dang, Y.H., et al. (2013). Roles of levo-tetrahydropalmatine in modulating methamphetamine reward behavior. *Physiol Behav* 118**,** 195-200. doi: 10.1016/j.physbeh.2013.05.034.

Sun, C., Chen, Z., Wang, H., and Ding, K. (2018a). Tetrahydropalmatine Prevents High-Fat Diet-Induced Hyperlipidemia in Golden Hamsters (Mesocricetus Auratus). *Med Sci Monit* 24**,** 6564-6572. doi: 10.12659/msm.910578.

Sun, R., Song, Y., Li, S., Ma, Z., Deng, X., Fu, Q., et al. (2018b). Levo-tetrahydropalmatine Attenuates Neuron Apoptosis Induced by Cerebral Ischemia-Reperfusion Injury: Involvement of c-Abl Activation. *J Mol Neurosci* 65(3)**,** 391-399. doi: 10.1007/s12031-018-1063-9.

Sun, S.J., Wei, Y.F., Wang, H., Tang, L.F., and Deng, B.Y. (2020). Simultaneous Electrochemiluminescence Determination of Sinomenine, Cepharanthine and Tetrahydropalmatine in Stephania epigaea by Capillary Electrophoresis Coupled with Ultrasonic-Assisted Aqueous Two-Phase Extraction. *International Journal of Electrochemical Science* 15(6)**,** 5002-5017. doi: 10.20964/2020.06.33.

Sushchyk, S., Xi, Z.X., and Wang, J.B. (2016). Combination of Levo-Tetrahydropalmatine and Low Dose Naltrexone: A Promising Treatment for Prevention of Cocaine Relapse. *J Pharmacol Exp Ther* 357(2)**,** 248-257. doi: 10.1124/jpet.115.229542.

Tao, Y., Huang, S., Yan, J., and Cai, B. (2019). Establishment of a rapid and sensitive UPLC-MS/MS method for pharmacokinetic determination of nine alkaloids of crude and processed Corydalis turtschaninovii Besser aqueous extracts in rat plasma. *J Chromatogr B Analyt Technol Biomed Life Sci* 1124**,** 218-225. doi: 10.1016/j.jchromb.2019.06.018.

Tao, Y., Jiang, E., and Cai, B. (2020). A biochemometrics strategy combining quantitative determination, bioactivity evaluation and relationship analysis for identification of analgesic alkaloids of raw and vinegar-processed Corydalis turtschaninovii. *J Sep Sci* 43(6)**,** 1183-1189. doi: 10.1002/jssc.201901021.

Wang, N., Xu, P., Wu, C., Wu, R., and Shou, D. (2019). Preparation of micro-cell membrane chromatographic columns with polyvinyl alcohol-modified polyether ether ketone tube as cellular membrane carrier. *J Chromatogr B Analyt Technol Biomed Life Sci* 1104**,** 102-108. doi: 10.1016/j.jchromb.2018.11.014.

Wang, X., Zhao, R., Zhang, H., Zhou, M., Zhang, M., and Qiao, T. (2018). Levo-Tetrahydropalmatine Attenuates Progression of Abdominal Aortic Aneurysm in an Elastase Perfusion Rat Model via Suppression of Matrix Metalloproteinase and Monocyte Chemotactic Protein-1. *Med Sci Monit* 24**,** 652-660. doi: 10.12659/msm.906153.

Wang, Y., Wang, R.R., Sun, W., Lou, C., Yang, F., He, T., et al. (2021). Analgesic effect of dl-THP on inflammatory pain mediated by suppressing spinal TRPV1 and P2X3 receptors in rats. *Front Biosci (Landmark Ed)* 26(5)**,** 1-10. doi: 10.52586/4919.

Wang, Z.Z., and Peng, G.P. (2002). Study on extraction of summer-free components by supercritical CO2 extraction technology, China Journal of Chinese Materia Medica 27(008), 585-586.

Wen, H., Zhang, H., Wang, W., and Li, Y. (2020). Tetrahydropalmatine protects against acute lung injury induced by limb ischemia/reperfusion through restoring PI3K/AKT/mTOR-mediated autophagy in rats. *Pulm Pharmacol Ther* 64**,** 101947. doi: 10.1016/j.pupt.2020.101947.

Wu, C., Yan, R., Zhang, R., Bai, F., Yang, Y., Wu, Z., et al. (2013a). Comparative pharmacokinetics and bioavailability of four alkaloids in different formulations from Corydalis decumbens. *J Ethnopharmacol* 149(1)**,** 55-61. doi: 10.1016/j.jep.2013.05.043.

Wu, C., Yan, R., Zhang, R., Fan, B., and Wu, A. (2013b). Comparative pharmacokinetics and bioavailability of four alkaloids in different formulations from Corydalis decumbens. *Journal of Ethnopharmacology* 149(1)**,** 55-61.

Wu, D.J.,Wu, J.G., and Wu, J.Z. (2011). Preparative isolation of dl-tedrahypalmatine and protopine from Corydalis racemosa(Thunb.)Pers.by high-speed counter-current chromatography, *Strait Pharmaceutical Journal* 23(10), 3.

Wu, L., Zhang, W., Qiu, X., Wang, C., Liu, Y., Wang, Z., et al. (2018). Identification of Alkaloids from Corydalis yanhusuo W. T. Wang as Dopamine D₁ Receptor Antagonists by Using CRE-Luciferase Reporter Gene Assay. *Molecules* 23(10). doi: 10.3390/molecules23102585.

Wu, L.L., Jiang, W.Z., Huang, X.Z., Shi, X.X., LV, X.X., Xu, C. Y., et al. (2011). Extraction and content determination of tetrahydropalmatine from Stephaniacepharantha Hayata, *Guangxi medical* (1), 09-111.

Xia, X., He, J., Liu, B., Shao, Z., Xu, Q., Hu, T., et al. (2020). Targeting ERα degradation by L-Tetrahydropalmatine provides a novel strategy for breast cancer treatment. *Int J Biol Sci* 16(12)**,** 2192-2204. doi: 10.7150/ijbs.44005.

Xiao, H.T., Peng, J., Liang, Y., Yang, J., Bai, X., Hao, X.Y., et al. (2011). Acetylcholinesterase inhibitors from Corydalis yanhusuo. *Nat Prod Res* 25(15)**,** 1418-1422. doi: 10.1080/14786410802496911.

Xiao, J., Pan, Y., Zhang, L., Wang, X., Han, Y., Sun, L., et al. (2019). High Performance Liquid Chromatography Determination and Optimization of the Extraction Process for the Total Alkaloids from Traditional Herb Stephania cepharantha Hayata. *Molecules* 24(3). doi: 10.3390/molecules24030388.

Xiao, J., Wang, Y., Yang, Y., Liu, J., Chen, G., Lin, B., et al. (2021). Natural potential neuroinflammatory inhibitors from Stephania epigaea H.S. Lo. *Bioorg Chem* 107**,** 104597. doi: 10.1016/j.bioorg.2020.104597.

Xu, Q.M., Liu, Y.L., Zhao, B.H., Xu, L.Z., Yang, S.L., and Chen, S.H. (2007). [Amides from the stems of Uvaria kweichowensis]. *Yao Xue Xue Bao* 42(4)**,** 405-407.

Xu,Z.K., Chen,G.B., Yin, H.M., Gao,J., Bi, Y.A., and X, W. (2015). Research on Extraction Technology of Corydalis yanhusuo, *Modernization of Traditional Chinese Medicine and Materia Medica-World Science a;nd Technology* (11), 4.

Yang, B.R., Yu, N., Deng, Y.H., Hoi, P.M., Yang, B., Liu, G.Y., et al. (2015). L-tetrahydropalamatine inhibits tumor necrosis factor-α-induced monocyte-endothelial cell adhesion through downregulation of intercellular adhesion molecule-1 and vascular cell adhesion molecule-1 involving suppression of nuclear factor-κ B signaling pathway. *Chin J Integr Med* 21(5)**,** 361-368. doi: 10.1007/s11655-015-2165-7.

Yu, J., Che, J., Liu, L., Yang, F., Zhu, X., and Cao, B. (2016). Tetrahydropalmatine attenuates irradiation induced lung injuries in rats. *Life Sci* 153**,** 74-81. doi: 10.1016/j.lfs.2016.03.056.

Yu, Q., Cheng, P., Wu, J., and Guo, C. (2021). PPARγ/NF-κB and TGF-β1/Smad pathway are involved in the anti-fibrotic effects of levo-tetrahydropalmatine on liver fibrosis. *J Cell Mol Med* 25(3)**,** 1645-1660. doi: 10.1111/jcmm.16267.

Yu, Q., Liu, T., Li, S., Feng, J., Wu, L., Wang, W., et al. (2018). The Protective Effects of Levo-Tetrahydropalmatine on ConA-Induced Liver Injury Are via TRAF6/JNK Signaling. *Mediators Inflamm* 2018**,** 4032484. doi: 10.1155/2018/4032484.

Yu, Q., Wu, L., Liu, T., Li, S., Feng, J., Mao, Y., et al. (2019). Protective effects of levo-tetrahydropalmatine on hepatic ischemia/reperfusion injury are mediated by inhibition of the ERK/NF-κB pathway. *Int Immunopharmacol* 70**,** 435-445. doi: 10.1016/j.intimp.2019.02.024.

Yue, K., Ma, B., Ru, Q., Chen, L., Gan, Y., Wang, D., et al. (2012). The dopamine receptor antagonist levo-tetrahydropalmatine attenuates heroin self-administration and heroin-induced reinstatement in rats. *Pharmacol Biochem Behav* 102(1)**,** 1-5. doi: 10.1016/j.pbb.2012.03.014.

Yun, J. (2014). L-tetrahydropalmatine inhibits methamphetamine-induced locomotor activity via regulation of 5-HT neuronal activity and dopamine D3 receptor expression. *Phytomedicine* 21(11)**,** 1287-1291. doi: 10.1016/j.phymed.2014.07.003.

Zhai, X.T., Chen, J.Q., Jiang, C.H., Song, J., Li, D.Y., Zhang, H., et al. (2016). Corydalis bungeana Turcz. attenuates LPS-induced inflammatory responses via the suppression of NF-κB signaling pathway in vitro and in vivo. *J Ethnopharmacol* 194**,** 153-161. doi: 10.1016/j.jep.2016.09.013.

Zhang, M.Y., Liu, Y.P., Zhang, L.Y., Yue, D.M., Qi, D.Y., Liu, G.J., et al. (2015). Levo-Tetrahydropalmatine Attenuates Bone Cancer Pain by Inhibiting Microglial Cells Activation. *Mediators Inflamm* 2015**,** 752512. doi: 10.1155/2015/752512.

Zhang, Y., Sha, R., Wang, K., Li, H., Yan, B., and Zhou, N. (2018). Protective effects of tetrahydropalmatine against ketamine-induced learning and memory injury via antioxidative, anti-inflammatory and anti-apoptotic mechanisms in mice. *Mol Med Rep* 17(5)**,** 6873-6880. doi: 10.3892/mmr.2018.8700.

Zhang, Y., Wang, Z., Xu, J., Yang, F., Dai, C., Xie, W., et al. (2020). Optimization of the extraction and purification of Corydalis yanhusuo W.T. Wang based on the Q-marker uniform design method. *BMC Chem* 14(1)**,** 9. doi: 10.1186/s13065-020-00666-6.

Zhao, B.H., Xu, Q.M., Zou, Z.M., Xu,L.Z., and Yang, S.L. (2006). Study on chemical constituents of aboveground part of Uvaria kweichowensis, *Chinese Traditional and Herbal Drugs* 37(5), 676-677.

Zhao, N., Chen, Y., Zhu, J., Wang, L., Cao, G., Dang, Y., et al. (2014). Levo-tetrahydropalmatine attenuates the development and expression of methamphetamine-induced locomotor sensitization and the accompanying activation of ERK in the nucleus accumbens and caudate putamen in mice. *Neuroscience* 258**,** 101-110. doi: 10.1016/j.neuroscience.2013.11.025.

Zhao, T., Liu, X., Zhen, X., and Guo, S.W. (2011). Levo-tetrahydropalmatine retards the growth of ectopic endometrial implants and alleviates generalized hyperalgesia in experimentally induced endometriosis in rats. *Reprod Sci* 18(1)**,** 28-45. doi: 10.1177/1933719110381928.

Zhao, Z.M., Shang, X.F., Lawoe, R.K., Liu, Y.Q., Zhou, R., Sun, Y., et al. (2019). Anti-phytopathogenic activity and the possible mechanisms of action of isoquinoline alkaloid sanguinarine. *Pestic Biochem Physiol* 159**,** 51-58. doi: 10.1016/j.pestbp.2019.05.015.

Zhi, X., Wang, L., Chen, H., Fang, C., Cui, J., Hu, Y., et al. (2020). l-tetrahydropalmatine suppresses osteoclastogenesis in vivo and in vitro via blocking RANK-TRAF6 interactions and inhibiting NF-κB and MAPK pathways. *J Cell Mol Med* 24(1)**,** 785-798. doi: 10.1111/jcmm.14790.

Zhou, H.H., Wu, D.L., Gao, L.Y., Fang, Y., and Ge, W.H. (2016). L-Tetrahydropalmatine alleviates mechanical hyperalgesia in models of chronic inflammatory and neuropathic pain in mice. *Neuroreport* 27(7)**,** 476-480. doi: 10.1097/wnr.0000000000000560.

Zhou, Z.Y., Zhao, W.R., Shi, W.T., Xiao, Y., Ma, Z.L., Xue, J.G., et al. (2019). Endothelial-Dependent and Independent Vascular Relaxation Effect of Tetrahydropalmatine on Rat Aorta. *Front Pharmacol* 10**,** 336. doi: 10.3389/fphar.2019.00336.
